# Localization of Nucleoporin Tpr to the Nuclear Pore Complex Is Essential for Tpr Mediated Regulation of the Export of Unspliced RNA

**DOI:** 10.1371/journal.pone.0029921

**Published:** 2012-01-13

**Authors:** Kalpana Rajanala, Vinay Kumar Nandicoori

**Affiliations:** National Institute of Immunology, Aruna Asaf Ali Marg, New Delhi, India; University of Cambridge, United Kingdom

## Abstract

Nucleoporin Tpr is a component of the nuclear pore complex (NPC) that localizes exclusively to intranuclear filaments. Tpr functions as a scaffolding element in the nuclear phase of the NPC and plays a role in mitotic spindle checkpoint signalling. Export of intron-containing mRNA in Mason Pfizer Monkey Virus is regulated by direct interaction of cellular proteins with the *cis*-acting Constitutive Transport Element (CTE). In mammalian cells, the transport of Gag/Pol-CTE reporter construct is not very efficient, suggesting a regulatory mechanism to retain this unspliced RNA. Here we report that the knockdown of Tpr in mammalian cells leads to a drastic enhancement in the levels of Gag proteins (p24) in the cytoplasm, which is rescued by siRNA resistant Tpr. Tpr's role in the retention of unspliced RNA is independent of the functions of Sam68 and Tap/Nxf1 proteins, which are reported to promote CTE dependent export. Further, we investigated the possible role for nucleoporins that are known to function in nucleocytoplasmic transport in modulating unspliced RNA export. Results show that depletion of Nup153, a nucleoporin required for NPC anchoring of Tpr, plays a role in regulating the export, while depletion of other FG repeat-containing nucleoporins did not alter the unspliced RNA export. Results suggest that Tpr and Nup153 both regulate the export of unspliced RNA and they are most likely functioning through the same pathway. Importantly, we find that localization of Tpr to the NPC is necessary for Tpr mediated regulation of unspliced RNA export. Collectively, the data indicates that perinuclear localization of Tpr at the nucleopore complex is crucial for regulating intron containing mRNA export by directly or indirectly participating in the processing and degradation of aberrant mRNA transcripts.

## Introduction

Nucleoporin Tpr was originally identified as the oncogenic activator of the met, raf, and trk protooncogenes [1], [2], [3]. Tpr is a large 270 kDa protein with a bipartite structure consisting of a ∼1600-residue α-helical coiled-coil N-terminal domain and a highly acidic noncoiled ∼800 amino acid carboxy terminus [4]. Cellular transformations and human tumors have been shown to occur due to the fusion of N-terminal residues of Tpr (residues 140–230) with the protein kinase domains of the protooncogenes met, raf, and trk [1], [2], [3]. Tpr has been shown to be localized exclusively to intranuclear filaments associated with the nucleoplasmic side of the NPC, by directly binding to Nup153 [5], [6], [7]. Different metazoan species have been shown to contain only one Tpr ortholog, whereas two homologs, Mlp1 and Mlp2, are present in *Saccharomyces cerevisiae* and *Schizosaccharomyces pombe* [8], [9], [10].

The functions of Tpr include roles in intranuclear and nucleocytoplasmic transport and as a scaffolding element in the nuclear phase of the NPC [11], [12], [13], [14]. Tpr has been shown to play a role in nuclear export of proteins containing leucine rich nuclear export signal (NES) and it also aids in the export of proteins with no apparent NES, as in the Huntington protein [12], [15]. The association of Mad1 and Mad2 proteins with Tpr has been shown to affect mitotic spindle checkpoint signalling [16]. Depletion of Tpr causes a chromosome lagging phenotype and this phenomenon is due to the loss of interaction between Tpr and dynein complex [17]. The Tpr homolog of Arabidopsis has been implicated in the regulation of mRNA export and SUMO homeostasis, and has been shown to influence various aspects of plant development like flowering time [18], [19]. The interaction between transcription factor HSF-1 and Tpr has been shown to facilitate the export of stress induced Hsp-70 mRNA [20]. In a previous study we have identified Tpr as a substrate of MAP kinase ERK2 and identified the ERK2 mediated phosphorylation sites on Tpr [21], [22]. Tpr has been demonstrated to act as an ERK2 scaffold in NPC, in turn resulting in phosphorylation of substrates that interact with Tpr.

Conventionally in eukaryotes, unspliced RNA is retained in the nucleus, and only processed mRNA is exported through the NPC. However, retroviruses have developed mechanisms to overcome this regulation, thus enabling unspliced genomic RNA to be exported and finally packaged. These mechanisms can be classified into two types, Rev dependent and Rev independent. The Rev dependent pathway, employed by the Human Immuno deficiency Virus (HIV), utilizes retroviral Rev protein bound to the Rev response element (RRE) [3] present in the unspliced transcripts [23], [24]. Once bound to RRE, Rev recruits host cellular factors such as Exportin-1 [25] and Sam68 (Src associated in Mitosis 68), to effect nuclear export through NPC. Sam68, a member of the STAR (Signal Transduction and Activation of RNA) family of proteins is functionally regulated by nuclear kinases SIK/BRK [26]. The Rev independent mechanism to export unspliced RNA is used in Mason Pfizer Monkey Virus (MMPV). This is mediated by a *cis* – acting element present in the unspliced transcript, known as the Constitutive Transport Element (CTE) [27], which directly recruits host cellular factors for exporting intron-containing RNA.

In the present study we report the results of a comprehensive analysis of nucleoporin Tpr's role in modulating protein import/export, mRNA export and the export of unspliced RNA. We find that Tpr does not seem to play any significant role in regulating protein import/export and mRNA export. However, it plays a definitive role in modulating CTE- mediated export of intron-containing RNA. Depletion of Tpr results in the enhancement of CTE function ensuring an increase in the export of Gag/Pol-CTE RNA, thus leading to a subsequent proportional rise in the Gag/Pol protein levels. Our data indicates that Tpr is a novel modulator of unspliced RNA export in mammalian cells, and its function is independent of those proteins which are known to promote CTE- mediated export of unspliced RNA. The results of our study clearly establish the importance of Tpr's localization at the NPC in facilitating the regulation of export of unspliced RNA in mammalian cells.

## Results

### Tpr does not play a significant role in cellular protein transport or in mRNA export

**Figure 1:**
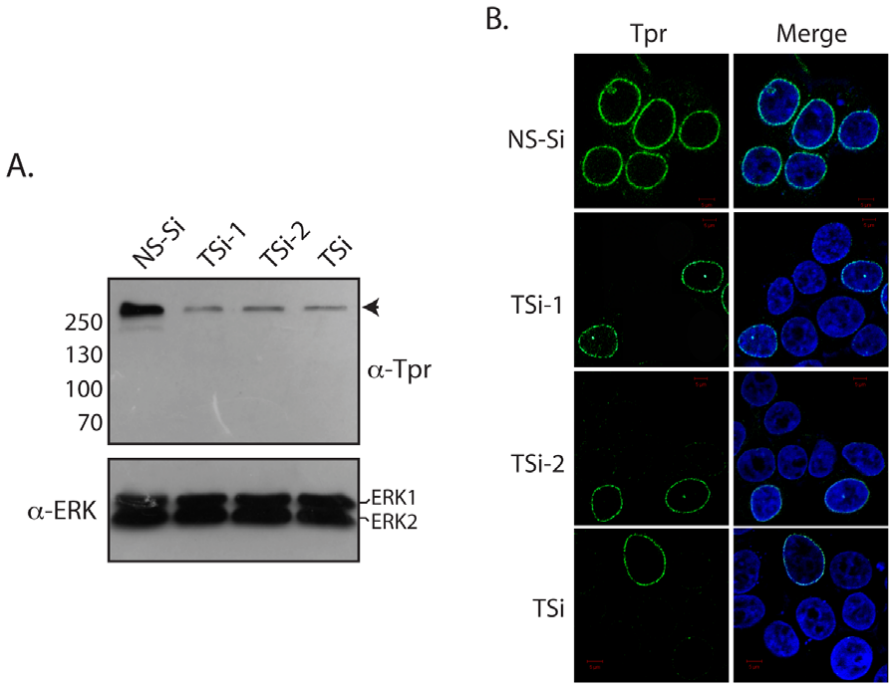
Tpr knockdown in HEK293T cells mediated by RNA interference. (A) Western blot analysis of whole cell extracts made from cells after 48 hours after treatment either with Non-specific siRNA (NS-Si) or three different Tpr siRNA's (TSi, TSi-1, TSi-2), probed with anti-Tpr and anti-ERK antibodies. (B) Immunofluorescence analysis of cells 48 hours after transfection with various Tpr siRNA oligonucleotides. Clear staining of nuclear membrane is seen in cells treated with NS-Si whereas only traces of staining is detected in cells where Tpr is knocked down.

Nucleoporin Tpr has been reported to play a role in nuclear export of proteins containing leucine rich nuclear export signal, [12] and the ectopic expression of mammalian Tpr has also been reported to result in the accumulation of poly (A)^+^ RNA in the nucleus [28]. We sought to comprehensively investigate the role of Tpr in nucleocytoplasmic transport of macromolecules. In order to examine the function of Tpr in cellular transport of proteins and nuclear export of mRNA, we depleted Tpr protein in HEK293T cells using three independent siRNA oligonucleotides. When the levels of Tpr were analyzed 48 hours post transfection, diminution in Tpr levels could be seen with all three siRNA oligos (Fig. 1A). Indirect immunofluorescence microscopy using mouse monoclonal anti-Tpr antibodies corroborated these findings (Fig. 1B).

**Figure 2:**
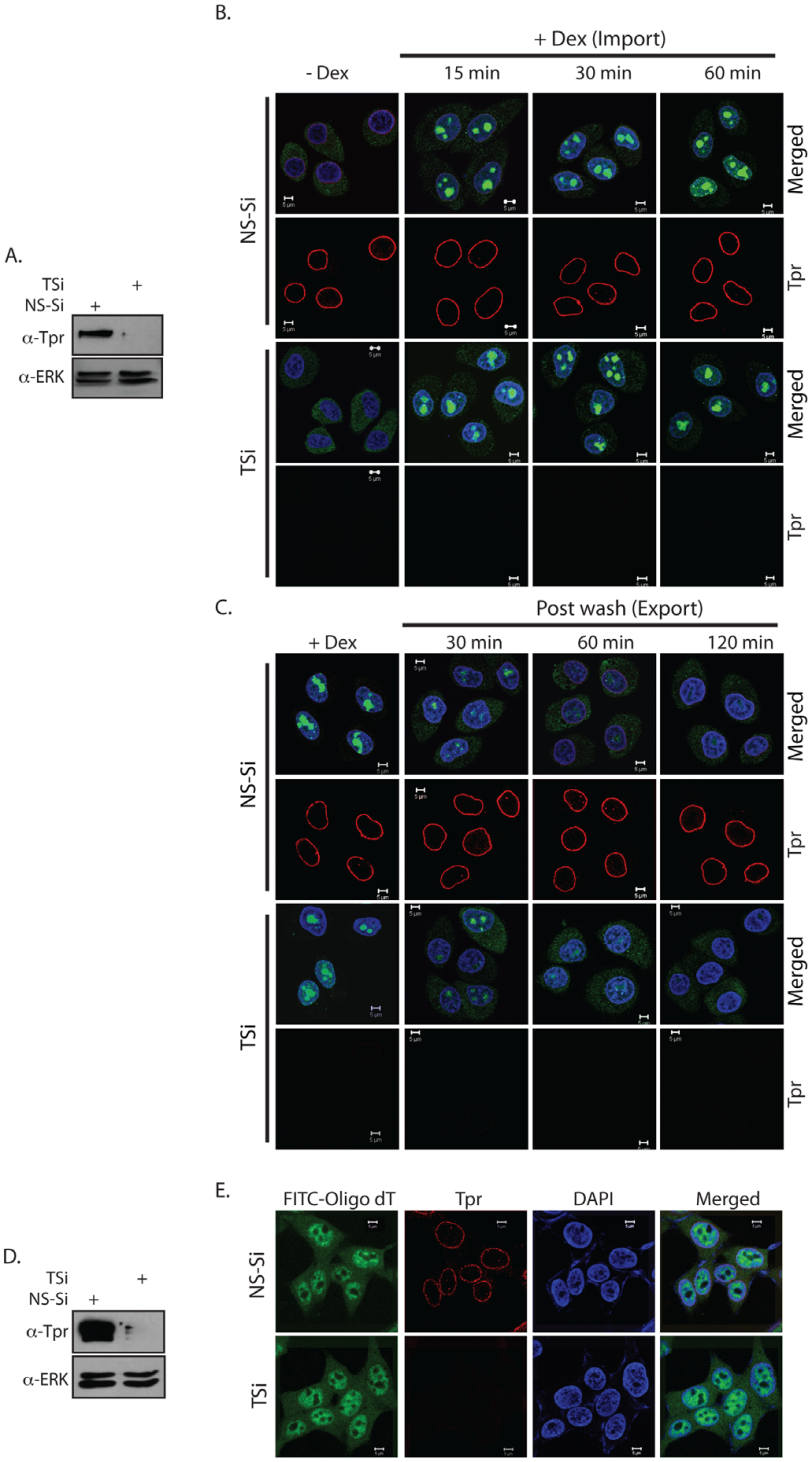
Depletion of Tpr has no significant effect on cellular protein transport and mRNA export. (A) Western blot analysis of extracts of HeLa cells made 48 hours after treatment with TSi. (B) HeLa cells which stably express hormone responsive GFP reporter construct (RGG) were transfected with NS-Si or TSi. Import of chimeric GFP was monitored by fixing the cells with 4% paraformaldehyde at different times post dexamethasone treatment. (C) Export of RGG construct was tracked at indicated time points after removal of the hormone. (D) Western blot depicting Tpr knockdown in HEK293T cells after 48 hours after siRNA transfection. (E) Fluorescence in situ hybridization with FITC-oligo(dT) probe to assess distribution of poly(A)^+^ mRNA in HEK293T cells transfected with TSi.

Love et al., [29] have established an exceedingly useful system to investigate import and export of proteins in HeLa cells. In these cells, chimeric Rev- Glucocorticoid-GFP Receptor protein (chimeric GFP) is localized to the cytosol in the absence of any treatment, and upon the addition of dexamethasone it translocates into the nucleus. Subsequent to the removal of dexamethasone, the chimeric GFP is exported back to the cytoplasm. Cells expressing this chimeric GFP protein were transfected with either the Non-Specific siRNA (NS-Si) or Tpr-siRNA (TSi) oligonucleotides and incubated for 48 hours to achieve the knockdown of Tpr protein (Fig. 2A). When import of the chimeric GFP protein was investigated by incubating the cells with dexamethasone, import rates were observed to be similar for both NS-Si and TSi treated cells (Fig. 2B), indicating absence of any role for Tpr in protein import. Subsequently, the export of the chimeric GFP-protein was visualized at different time intervals after the removal of dexamethasone. Interestingly, we observed slight, but reproducible, decrease in the rate of chimeric GFP protein export in the cells treated with TSi in comparison with those treated with NS-Si, at 30 and 60 min after washing off the drug/hormone. However, 2 h after the drug was removed, complete translocation of chimeric GFP to the cytosol was noticed in both cases (Fig. 2C). These results indicate that Tpr may have a limited role in modulating the rate of protein export. To investigate the role of Tpr in export of processed poly (A)^+^ mRNA, we knocked down Tpr expression (Fig. 2D) and performed Fluorescence in situ hybridization using FITC tagged oligo(dT) probe (Fig. 2E). The distribution of poly (A)^+^ mRNA was found to be comparable in both control and in Tpr depleted cells (Fig. 2E). Based on these results, we conclude that Tpr does not play a role in translocation of proteins or poly (A)^+^ mRNA across the nuclear membrane.

### Tpr modulates CTE dependent export of unspliced RNA

**Figure 3:**
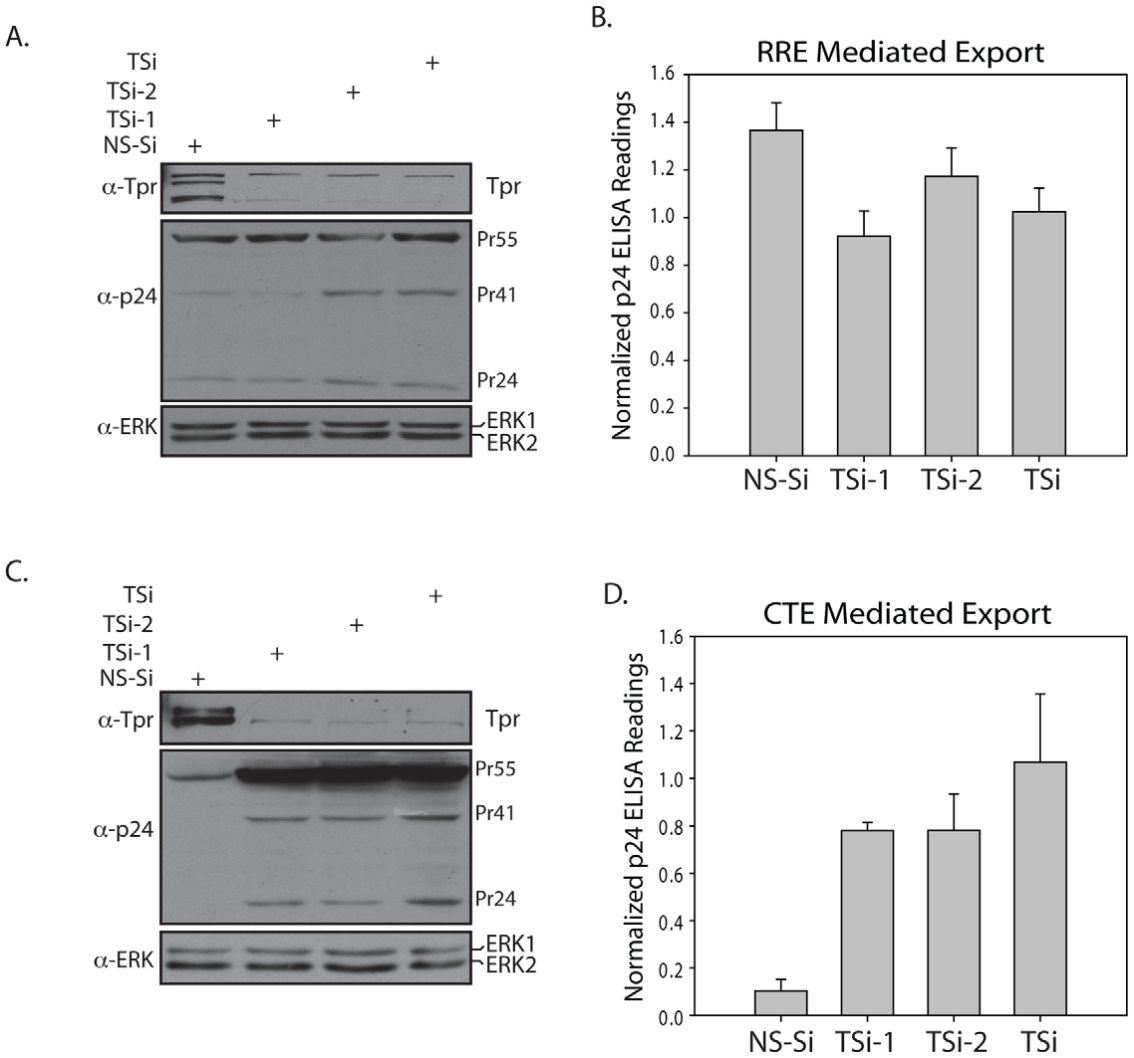
Elevated p24 levels resulting from enhanced CTE function are observed in Tpr knockdown cells. (A) HEK293T cells were transfected with either NS-Si or with different siRNA oligos against Tpr along with 100 ng of Gag/PR-RRE, 75 ng of pcDNA3-Flag-Rev and 100 ng of CMV- β-Gal constructs. 48 hours post transfection the lysates were resolved on SDS-PAGE, transferred on to nitrocellulose membrane and probed with anti-Tpr, anti-ERK and anti-p24 antibodies. (B) p24 and β-Galactosidase levels in each of the samples were assayed. The p24 values were then adjusted against variations in β-Gal readings and the normalized values are presented here. Average values of three independent transfections are presented; the error bars represent the standard deviation. (C) Western blot of HEK293T cell extracts depicting elevated levels of Gag/pol protein cleavage products in cells transfected with Gag/Pol-CTE reporter construct, CMV- β-Gal and different Tpr siRNA's. (D) Effect of Tpr knockdown on p24 expression from Gag/pol-CTE reporter as demonstrated by p24 ELISA readings after normalization against β-Galactosidase levels.

Tpr homologs in *S.cerevisiae*, Mlp1 and Mlp2, were reported to play a role in export of unspliced RNA [30]. Towards analyzing the role of Tpr in the nucleocytoplasmic export of intron-containing RNA, we utilized well characterized Gag/Pol reporter constructs containing either a Rev response element (RRE) [3] or a constitutive transport element (CTE) [26], [31], [32], [33], [34]. These reporter constructs contain coding sequences of Gag/Pol proteins of HIV within an intron, followed by either RRE or CTE. We first examined the possibility of a role for Tpr in RRE-dependent export. Western blot analysis showed that when Tpr was indeed knocked down there was negligible variation in the Gag protein cleavage products p55, p41 and p24 (Fig. 3A). The levels of Gag protein in the cytoplasm were quantified with the help of p24 ELISA, which measures the amount of processed Gag protein (p24). It is apparent from the data presented (Fig. 3B, Fig. S1A and S1B), that the levels of p24 in the extracts remained the same, irrespective of Tpr protein levels, indicating that Tpr does not play any role in RRE-Rev dependent export of unspliced RNA.

**Figure 4:**
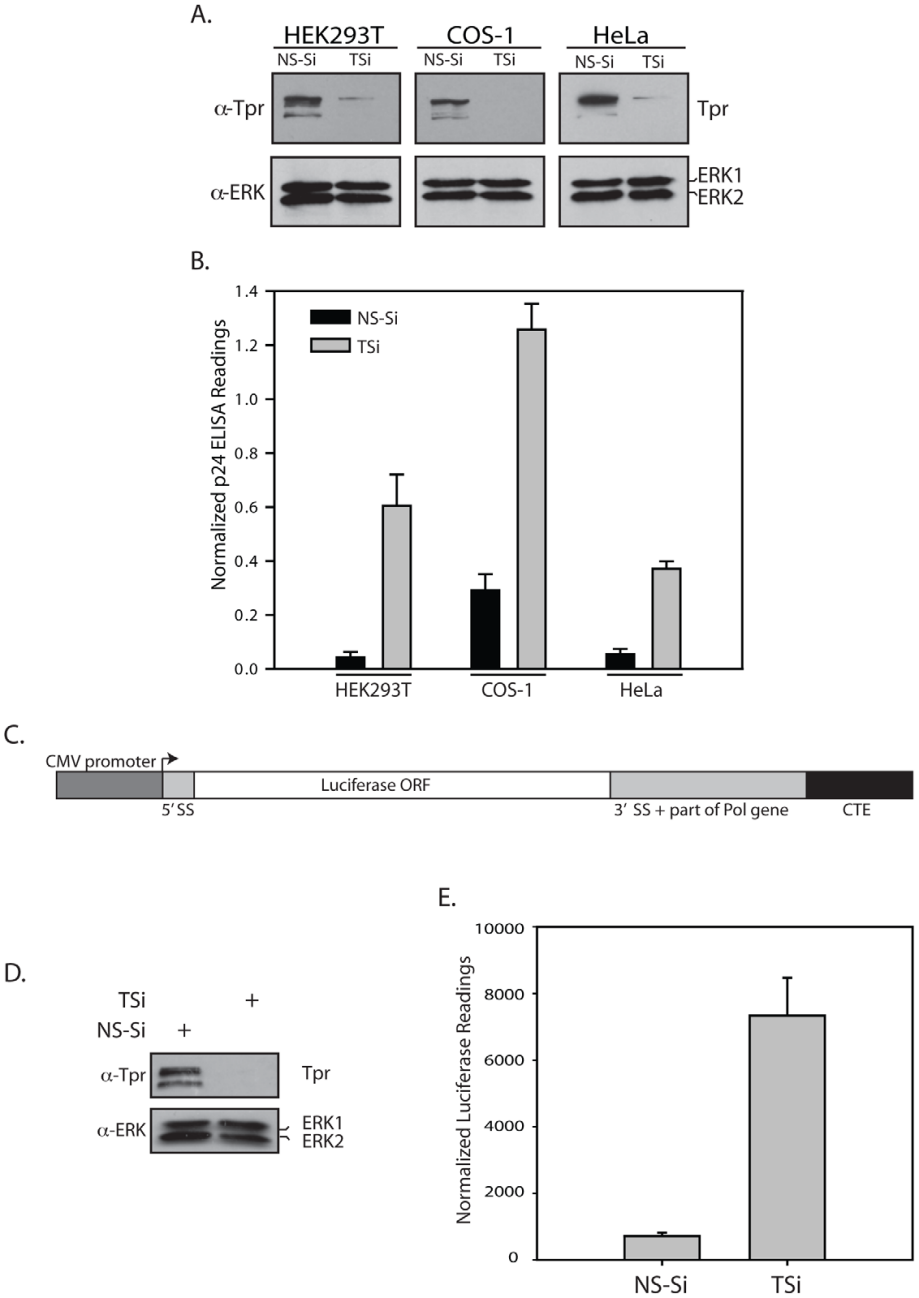
Depletion of mammalian Tpr significantly enhances CTE function. (A) HEK293T, COS-1 and HeLa cells were transfected with either NS-Si or TSi along with Gag/Pol-CTE reporter construct, and the lysates were probed with anti-Tpr, anti-ERK antibodies. (B) The cell lysates were assayed for p24 and β-Gal expression and the p24 levels were normalized using β-Gal readings. (C) Schematic representation of the Luciferase-CTE (Luc-CTE) reporter construct. (D) Western blot analysis of extracts of HEK293T cells co-transfected with NS-Si or TSi, CTE-Luc and CMV- β-Gal, to examine Tpr knockdown 48 hours after transfection. (E) The luciferase activity in the samples was recorded and the readings were adjusted against variations in β-Gal readings.

We then investigated the possible role of Tpr in regulating CTE-dependent export of unspliced RNA. Western blot analysis of extracts made from cells treated with Tpr siRNA showed that decrease in Tpr expression significantly increases levels of Gag protein cleavage products p55, p41 and p24 (Fig. 3C). We observed an ∼8–10 fold increase in the normalized p24 levels in the extracts prepared from TSi transfected cells compared with the NS-Si transfected cells (Fig. 3D, Fig. S1C and S1D). Next, we sought to investigate if the phenomenon of Tpr mediated regulation of unspliced RNA export can be observed in cells other than HEK293T. Results demonstrated enhanced CTE function upon Tpr depletion in COS-1 and HeLa cell lines (Fig. 4A and 4B, Fig. S2A and S2B). Though the fold increase in the export of unspliced RNA is different for different cell lines, the key finding that Tpr plays a role in retention of unspliced RNA in the nucleus could be consistently observed in all three cell lines. In order to rule out the possibility that the detected increase in nuclear export of reporter gene is limited to *gag/pol* gene, we created a Luciferase (Luc) reporter construct, in which the coding sequence of the *luc* gene was sandwiched between 5′ and 3′ splice site sequences originating from the HIV *gag/pol* gene followed by CTE (Fig. 4C). As evident from Figure 4, diminution of Tpr levels (Fig. 4D) resulted in ∼6–8 fold increase in the normalized luciferase activity (Fig. 4E, Fig. S2C and S2D). Taken together, these results suggest a regulatory role for Tpr in modulating CTE-dependent export of unspliced RNA.

### Ectopic and stable expression of siRNA resistant Tpr rescues CTE mediated unspliced RNA export

**Figure 5:**
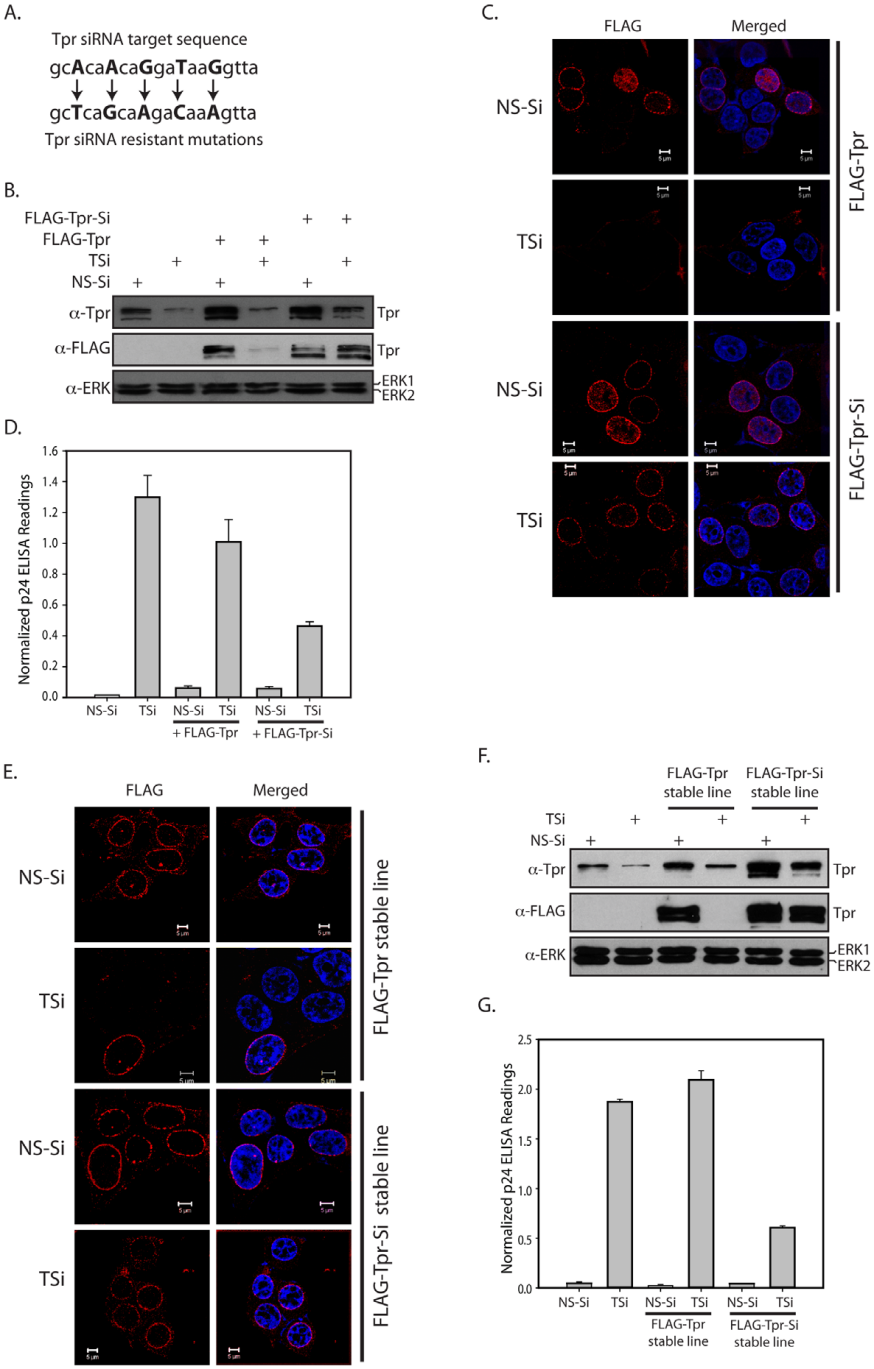
Ectopic and stable expression of siRNA resistant Tpr rescues CTE mediated unspliced RNA export. (A) Schematic representation of the silent point mutations that were introduced into the nucleotide sequence of Tpr, to protect it from being targeted by siRNA duplexes. (B) Immunoblot demonstrating the restoration of Tpr levels upon co- transfection of FLAG-Tpr-Si construct with TSi. 2 µg of Flag-Tpr or Flag-Tpr-Si were used for transfection. (C) Indirect immunofluorescence microscopy of HEK293T cells transfected with Wild-type Flag-Tpr clone or siRNA resistant clone of Tpr along with NS-Si or TSi using mouse monoclonal anti-Flag antibodies (D) p24 and β-Galactosidase levels in each of the samples were analyzed. The p24 values were then adjusted against variations in β-Gal readings. (E) HEK293T cells stably expressing Flag-Tpr or Flag-Tpr-Si were transfected with either NS-Si or TSi together with Gag/Pol-CTE and CMV- β-Gal plasmids. Western blot of whole cell extracts probed with anti-Tpr, anti-FLAG and anti-ERK antibodies depicting stable expression. (F) Immunofluorescence analysis of Flag-Tpr or Flag-Tpr-Si stable cells after 2 days of transfection with either NS-Si or TSi oligonucleotides. (G) The cell lysates were assayed for p24 and β-Gal expression and the p24 levels were normalized with β-Gal readings.

To reinforce our findings we attempted to rescue the phenotype of increased export of unspliced RNA by co-transfecting cells with a plasmid containing siRNA-resistant *tpr* gene and TSi oligonucleotide. In order to generate an siRNA resistant Tpr clone, we have introduced silent point mutations into the wobble positions in the TSi's target sequence by overlapping PCR (Fig. 5A). As expected, in the cells co-transfected with TSi and FLAG-Tpr construct the levels of the Tpr detected are similar to those observed in TSi transfected cells (Fig. 5B; compare lane 4 with 2). However, when the cells are co-transfected with TSi and FLAG-Tpr-Si construct, the Tpr levels detected were almost equivalent to control (Fig. 5B; compare lane 6 with 1), indicating that the rescue plasmid is indeed resistant to siRNA. Indirect immunofluorescence microscopy using mouse monoclonal anti-Flag antibodies indicated that the efficiency of transfection is ∼40–60% (Fig. 5C and Figs. S3A and S6C). Similar to the results in Figure 3, we observed significant increase in the levels of normalized p24 when the intracellular Tpr was knocked down using TSi, which predictably did not alter when the cells were co-transfected with FLAG-Tpr (Fig. 5D, Fig. S3B and S3C). We observed an almost threefold reduction in the p24 levels when Tpr levels were restored by co-transfecting the cells with the siRNA resistant construct. However, even with repeated experimentation, the levels of the normalized p24 did not lower to control levels. This is most likely due to the fact that all Tpr knockdown cells do not express FLAG-Tpr-Si and the expression levels varied from cell to cell (Fig. 5C). In order to address this problem, we generated HEK293T stable cell lines expressing either the wild-type FLAG-Tpr or FLAG-Tpr-Si. Indirect immunofluorescence data suggests similar and uniform expression of FLAG-Tpr and FLAG-Tpr-Si constructs (Fig. 5E). Importantly, when the lysates were probed with FLAG antibodies we observed the expression levels of FLAG tagged Tpr in stably transfected cells to be almost equivalent (Fig. 5F). The normalized p24 levels resulting from Tpr depletion in Flag-Tpr stable cell line were similar to those obtained with parental 293T cells (Fig. 5G). When Flag-Tpr-Si stable cells were transfected with either NS-Si or TSi and Gag/Pol-CTE, we observed almost fourfold reduction in the p24 levels compared to FLAG-Tpr stable cell line (Fig. 5G and Fig. S3D and S3E). These findings show that the regulation of CTE mediated p24 gene expression can be specifically attributed to the alterations of Tpr levels in HEK293T cells.

### Tpr depletion enhances CTE mediated export of unspliced RNA from the nucleus

**Figure 6:**
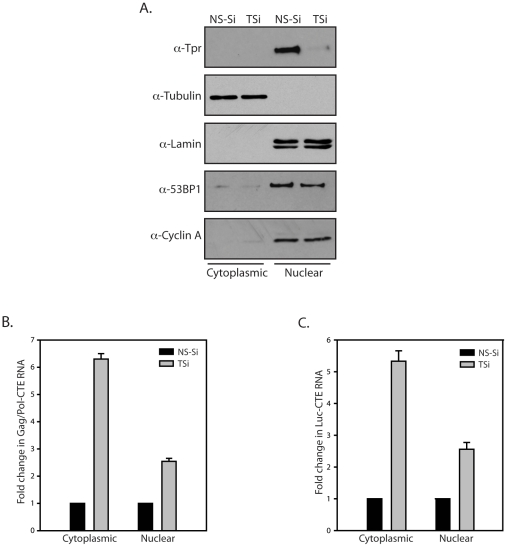
Tpr Depletion enhances CTE mediated unspliced RNA export from the nucleus. (A) Western blot analysis of cytosolic and nuclear fractions using anti-Tubulin, anti-Lamin, anti-Tpr, anti-53BP1 and anti-CyclinA antibodies. (B) Real-Time PCR analysis of mRNA isolated from nuclear and cytoplasmic fractions of HEK293T cells co-transfected with Gag/Pol-CTE construct and NS-Si or TSi. (C) Luc-CTE RNA levels in the nucleus and cytoplasm of cells transfected with pcDNA3-Luc-CTE construct and NS-Si or TSi measured by real time PCR.

Increase in the normalized p24 levels can also be attributed to increased translation of *gag/pol* RNA in the cytosol. In order to ascertain if the Tpr dependent increase in the p24 protein levels is indeed due to the export of unspliced RNA, we performed quantitative real time PCR analysis. Nuclear and cytoplasmic fractionation was performed as described and RNA and protein fractions were prepared from the extracts. Western blotting analysis (Fig. 6A) clearly showed presence of Tpr in nuclear fraction and tubulin in the cytoplasmic fraction. Further, soluble nuclear proteins 53BP1, CyclinA and the nuclear marker Lamin could be detected only in the nuclear fraction indicating the fractions obtained were free of any contamination. Real time PCR analysis established that the cytoplasmic unspliced Gag/Pol-CTE RNA was significantly higher in the Tpr depleted cells compared with NS-Si treated cells, suggesting increased export of unspliced RNA transcripts (Fig. 6B). Similar fold change in the nuclear and cytoplasmic mRNA levels was also observed in case of unspliced Luc-CTE RNA (Fig. 6C). The increase in the cytoplasmic unspliced RNA levels is reflected in the proportionate increase in the p24 levels shown in Figures 3, 4, and 5, reinforcing the fact that Tpr regulates CTE-mediated export of intron containing mRNA. To our surprise, we found that nuclear CTE mRNA levels were also ∼2–3 fold higher in the absence of Tpr (Fig. 6B and 6C). Yeast homologs of Tpr, the Mlp proteins have been shown to downregulate the expression of genes in response to the presence of aberrant RNA transcripts [35]. We speculate that the increase in the CTE-mRNA pools in the nucleus could be due to increased expression of the CTE-mRNA transcripts in Tpr depleted cells and/or increased stability of these transcripts in the nucleus when Tpr is knocked down. It is possible that in addition to regulating the export of unspliced RNA by causing them to be retained in the nucleus, Tpr may play a role in modulating the processing of mRNA molecules before the transcripts are actually exported.

### Tpr mediated regulation of unspliced RNA export is independent of Sam68 and Tap functions

**Figure 7:**
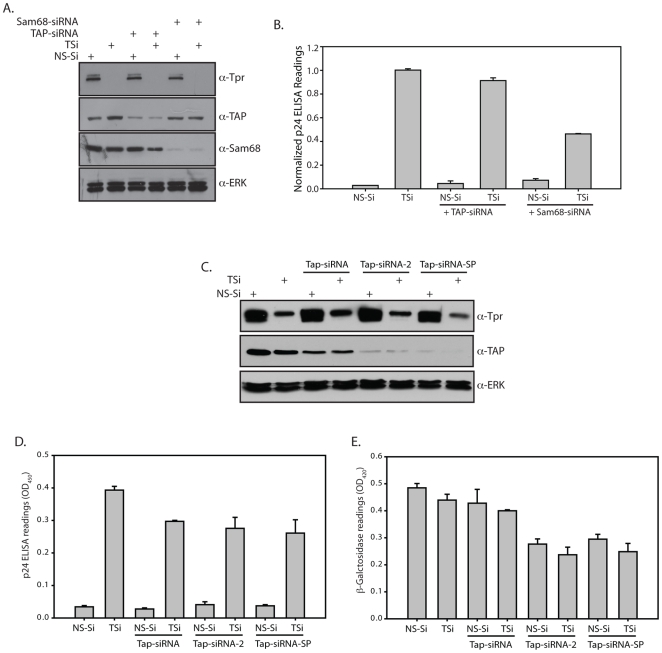
Tpr mediated regulation of unspliced RNA export is independent of Tap/Nxf1 and Sam68's functions. (A) Cells were transfected with 1 µg of siRNA against either Sam68 or Tap/Nxf1 along with NS-Si or TSi. The cells were replated the next day and 24 hours after replating, the cells were transfected once again with siRNA oligos and the reporter constructs. Western blot analysis of extracts of transfected HEK293T cells confirming the depletion of Tpr, Sam68 and Tap/Nxf1 proteins on treatment with respective siRNA's. (B) 48 hours after the second transfection, the amount of reporter gene expression from transfected Gag/Pol-CTE and CMV- β-Gal constructs was assayed. The p24 ELISA readings obtained from the samples were adjusted against variations in the β-Galactosidase readings. (C) Cells were transfected with 1 µg of different siRNA oligos against Tap/Nxf1 along with NS-Si or TSi. The cells were replated the next day and 24 hours after replating, the cells were transfected once again with siRNA oligos and the reporter constructs. Western blot of extracts of transfected HEK293T cells to analyze the depletion of Tpr and Tap/Nxf1 proteins. (D & E) The amount of reporter gene expression from transfected Gag/Pol-CTE and CMV- β-Gal constructs were assayed.

Previous reports have demonstrated a critical role for Sam68 and Tap proteins in the enhancement of CTE function by increasing the stability and utilization of unspliced RNA [26], [34]. In an effort to understand the mechanism behind Tpr mediated regulation, we studied the effect of Sam68 and Tap depletion as well as overexpression on CTE mediated export in Tpr deficient cells. HEK293T cells were co-transfected with Gag/Pol-CTE and CMV- β-Gal reporter constructs along with NS-Si or TSi in combination with either Tap-siRNA or Sam68-siRNA and analyzed 96 hours after siRNA treatment in order to obtain efficient knockdowns. Western blot analysis of the lysates showed decrease in the protein levels of Tpr and Sam68 in the response to treatment with their respective siRNA (Fig. 7A). However, the depletion of Tap upon siRNA treatment was not very efficient (Fig. 7A). Analysis of normalized p24 in the cells lysates showed that depletion of Tap or Sam68 by themselves do not greatly influence the export of unspliced RNA (Fig. 7B, Fig. S4A and S4B). Interestingly, depletion of Tpr and Sam68 together resulted in decreased p24 in the cytosol. This may be due to the fact that Sam68 is known to be required for stabilization of unspliced RNA [36], which may have been compromised to an extent in its absence. Tap/Nxf1 has been shown to have an effect on the export of mRNA from the nucleus in various model systems [37], [38], [39], [40]. Yet we did not observe noticeable decrease upon partial depletion of Tap either in p24 or β-GAL levels (Fig. S4A and S4B). Differences between the earlier Tap siRNA studies and our results could be due to the differences in the efficiency of siRNA in knocking down the Tap expression, as the knockdown detected in our experiment is not as efficient as those presented in the earlier reports. In order to address this concern, we repeated the experiment including two additional siRNAs, one an alternate siRNA (Tap-siRNA-2) and the second, a pool of four different siRNAs (Tap-siRNA-SP), to deplete Tap/Nxf1 protein. While the knockdown obtained with Tap-siRNA was similar to our earlier observation, both Tap-siRNA-2 and Tap-siRNA-SP were quite effective in depleting Tap protein (Fig. 7C). We also observed that the recovery of cells was lower after the treatment with the newer siRNAs compared to the cells transfected with Tap-siRNA. Consistent with the previous studies, when we depleted Tap with siRNA2 and siRNA-SP, we observed significant decrease in the β-galactosidase activity in the lysates (∼50%; Fig. 7E). Since the β-galactosidase activity was affected by the TAP depletion, we have not normalized the p24 readings. Interestingly, we did not detect substantial reduction in the p24 levels, indicating that the export of unspliced RNA seems to be independent of TAP depletion (Fig. 7D).

**Figure 8:**
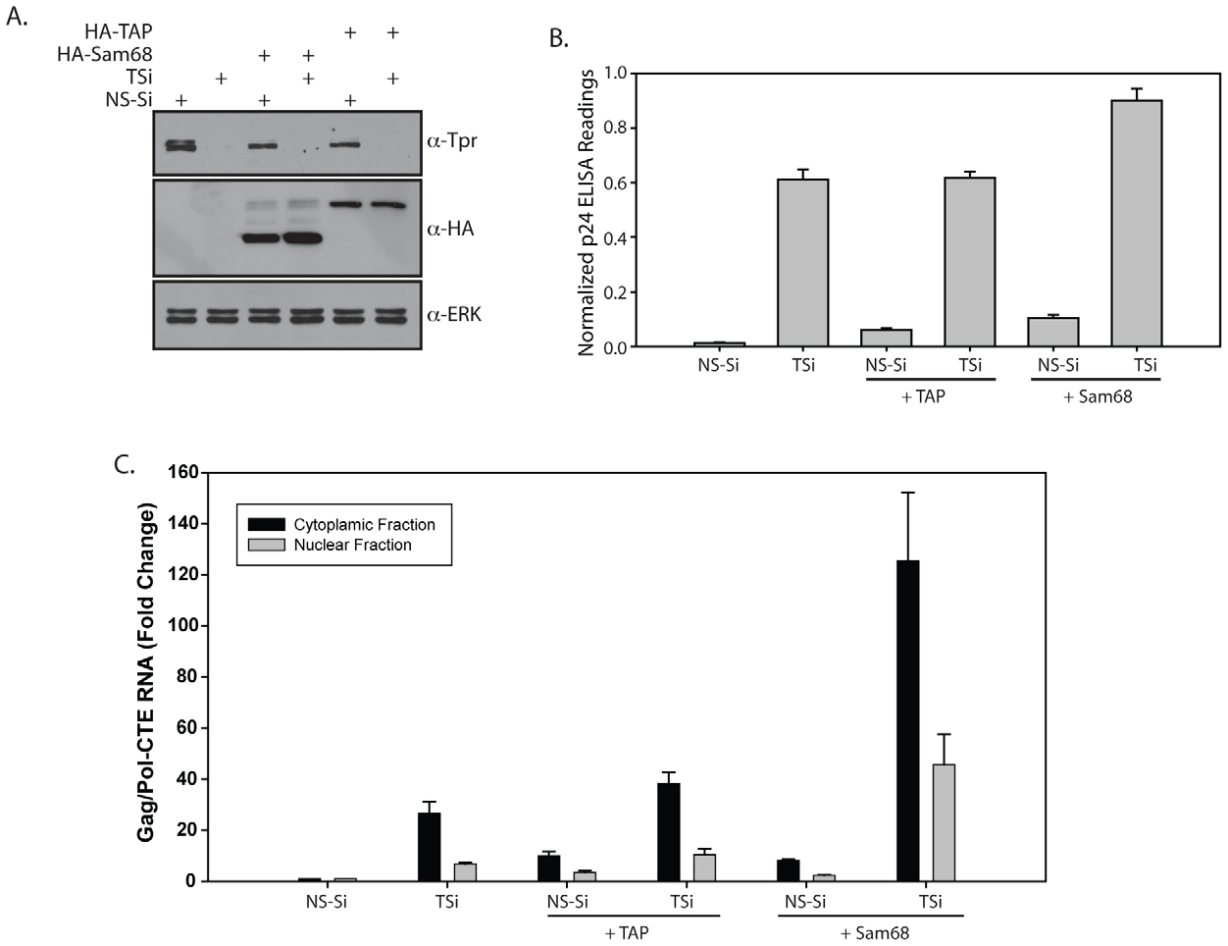
Overexpression of Sam68 in Tpr depleted cells leads to synergistic increase in the unspliced RNA export. (A) 48 hours after transfection with NS-Si and TSi, HEK293T cells were re-transfected with the same siRNA oligos along with Gag/Pol-CTE and CMV- β-Gal plasmids and 2 µg of HA-Sam68 or HA-Tap/Nxf1constructs. The lysates thus obtained were analyzed by Immunoblot for knockdown of Tpr and overexpression of HA-Sam68 and HA-Tap (B) The amount of p24 and β-Gal expression was estimated in the lysates and the obtained values were normalized. (C) Real-Time PCR analysis of mRNA isolated from nuclear and cytoplasmic fractions of HEK293T cells co-transfected with either NS-Si or TSi along with HA-Sam68 or HA-Tap/Nxf1constructs and Gag/Pol-CTE reporter plasmid.

Overexpression of Sam68 has been shown to enhance the stability and utilization of unspliced RNA, thus resulting in increased p24 levels [26]. Tap overexpression has been shown to improve the cytoplasmic utilization of Gag/Pol-CTE mRNA [34]. If Tpr depletion and Sam68 overexpression were to enhance the export in an inter-dependent manner, a combinatorial effect of depletion and overexpression would not be more than the independent effects. However, if they were to modulate the process independently, one would expect a cumulative effect. To address these two possibilities, we overexpressed either HA-Sam68 or HA-Tap in the cells transfected with either NS-Si or TSi and measured the normalized p24 levels in the extracts. Western blot data depicted in Figure 8A shows effective depletion of Tpr and overexpression of HA-Tap and HA-Sam68. In the samples where Tap or Sam68 were overexpressed in presence of NS-Si, we detected increase in the normalized p24 in the extracts as compared to NS-Si only (Fig. 8B). Depletion of Tpr by itself had significant effect on p24 levels in the extract (Fig. 8B). Interestingly, in the sample where Tap was overexpressed in the presence of TSi, we did not observe any further increase (Fig. 8B, Fig. S4C and S4D). In contrast, when Sam68 was overexpressed in Tpr depleted cells, we observed a modest synergistic increase in the p24. Further, analysis of Gag/Pol-CTE RNA levels in these samples by quantitative real time PCR demonstrated the same trend (Fig. 8C). Depletion of Tpr by itself increased the Gag/Pol RNA levels both in nucleus and cytoplasm, which did not substantially alter when Tap was overexpressed (Fig. 8C). This is not very surprising as Tap is shown to improve the cytoplasmic utilization of Gag/Pol-CTE RNA, which can only happen after its export [34]. However, when Sam68 was overexpressed in Tpr depleted samples, we observed significant increase in the Gag/Pol RNA levels (Fig. 8C), which was in turn reflected in increased p24 levels (Fig. 8B). Based on these results we believe that both Tpr and Sam68 regulate unspliced RNA export at different stages and a combinatorial effect of depletion and overexpression results in synergistic effect.

### Nucleoporin Nup153, a Tpr anchoring protein is also involved in regulating unspliced RNA export

**Figure 9:**
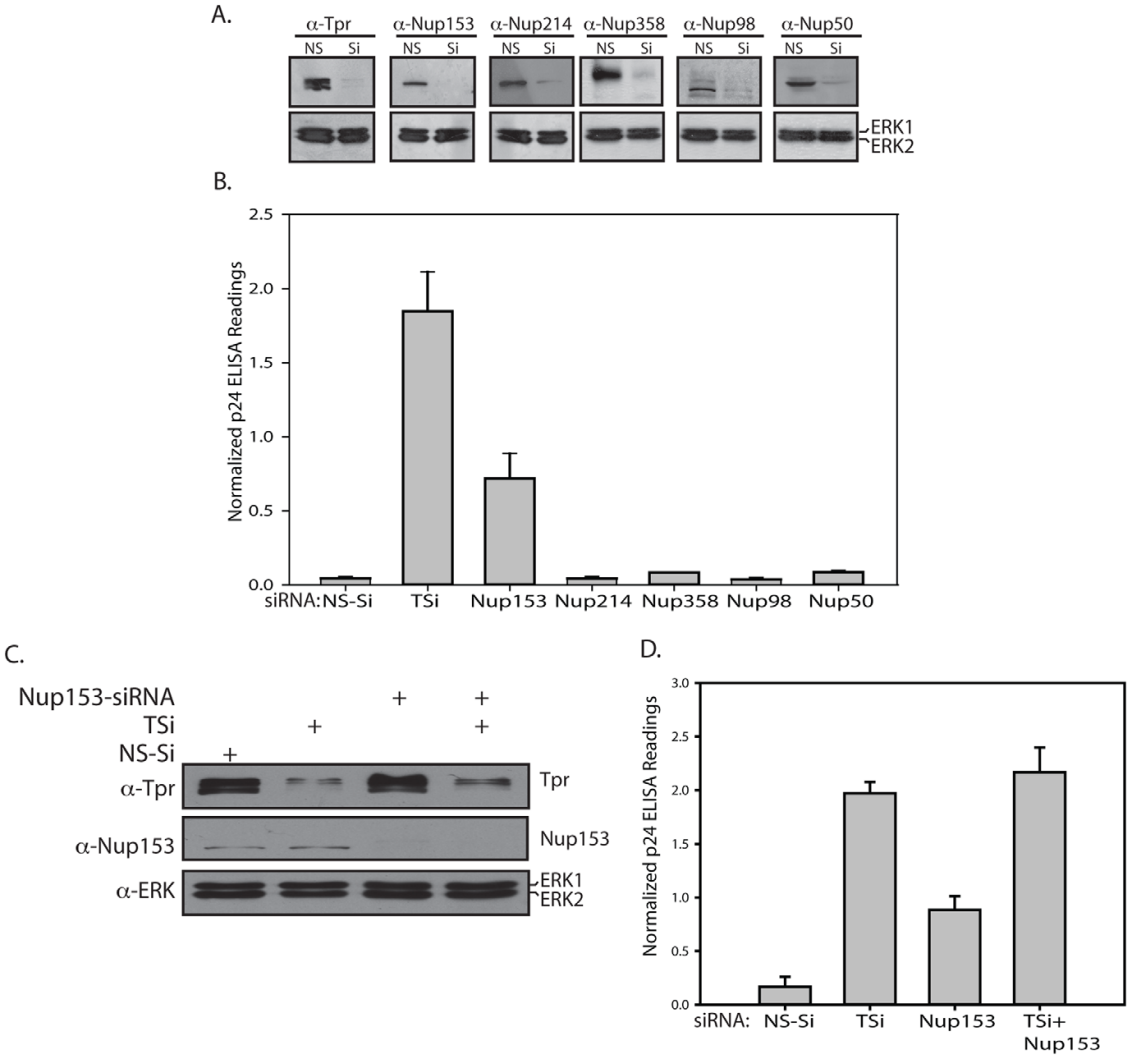
Nucleoporin Nup153, a Tpr anchoring protein is also involved in regulating unspliced RNA export. (A) HEK293T cells were transfected with siRNA's against different nucleoporins together with Gag/Pol-CTE and CMV- β-Gal constructs. Western blot of cell extracts confirming the depletion of various nucleoporins on treatment with respective siRNA's. (B) 48 hours post transfection, the amount of p24 and β-Gal expression was estimated in the lysates and the obtained values were normalized. (C) Cells were transfected with siRNA's against Nup153 or Tpr or Nup153+Tpr together with Gag/Pol-CTE and CMV- β-Gal constructs. Western blot of cell extracts confirming the depletion of Nup153 and Tpr on treatment with respective siRNA's. (D) The amount of normalized p24 expression was estimated in the lysates.

Our results thus far firmly established a role for Tpr in regulating CTE mediated unspliced RNA export. However, there exists the possibility that in addition to Tpr, other nucleoporins known to play a role in nucleocytoplasmic transport may also be involved in this modulation. Nucleoporins with conserved FXFG (phenylalanine and glycine) repeats in their sequence play a significant role in mediating nucleocytoplasmic transport by providing an interaction interface that aids in the translocation of receptor–cargo complexes through the NPC [41]. Various FG repeat-containing nucleoporins such as Nup214, Nup358/RanBP2, which are localized to the cytoplasmic fibrils of the NPC, and Nup153, Nup98 and Nup50, shown to be present in the nucleoplasmic side of the NPC, were considered for this study. Specific siRNAs were used to individually deplete the above mentioned nucleoporins (Fig. 9A) and the export of Gag/Pol-CTE RNA was quantitated by measuring the normalized p24 levels in the protein extracts. While we observed elevated levels of normalized p24 expression in samples when either Tpr or Nup153 were depleted, depletion of the other FG containing nucleoporins had no effect (Fig. 9B, Fig. S5A and S5B). Interaction between Tpr and Nup153 has been reported to be required for localization of Tpr to the nuclear pore [7]. If the increased export of Gag/Pol-CTE RNA observed upon Nup153 depletion is an independent function of Nup153, we would expect a cumulative increase when both Tpr and Nup153 are depleted. We observed enhanced p24 levels when either Tpr or Nup153 are depleted, with Tpr depletion enhancing p24 levels more effectively than Nup153 depletion (Fig. 9C and 9D, Fig. S5C and S5D). However, we did not observe a cumulative elevation of p24 levels when both of them were depleted, rather, we detected p24 levels similar to those observed for Tpr knockdown by itself. These results suggest that Tpr and Nup153 both regulate the export of unspliced RNA and they are most likely functioning through the same pathway.

### Localization of Tpr at the Nucleopore complex is necessary for the regulation of CTE mediated Export

In order to determine the region of Tpr that is necessary and sufficient for regulating export of unspliced RNA, we produced a series of siRNA resistant deletion constructs of Tpr (Fig. S6A). Results showed that only the N-terminal fragment and the Tpr-NM fragment containing both the N- and Middle regions was able to rescue the phenotype (Fig. S6B and S6C). Tpr has previously been shown to possess a nuclear localization signal in the carboxy terminal region, and the N-terminal fragment is reported to contain a NPC associating domain [28]. When we carried out immunofluorescence analysis to ascertain the subcellular localization of various Tpr fragments, we found TprN and NM to largely localize to the cytosol (Fig. S6E). However, we cannot rule out the possibility of at least some amounts TprN and TprNM may be able to associate with NPC even though they do not localize to the nuclear interior. Another possibility is that the overexpression of TprN and TprNM coupled to their largely cytosolic localization may result in other hitherto unkonwn cellular factors essential for CTE mediated unspliced RNA export and subsequent gene expression, being titrated out by TprN and TprNM. Further, we observed aberrant nuclear blebbing when TprN and TprNM are overexpressed, and this too may impact CTE mediated RNA export. These aspects need to be further investigated before any definitive conclusions may be drawn as some of the effects may be overexpression phenotypes.

**Figure 10:**
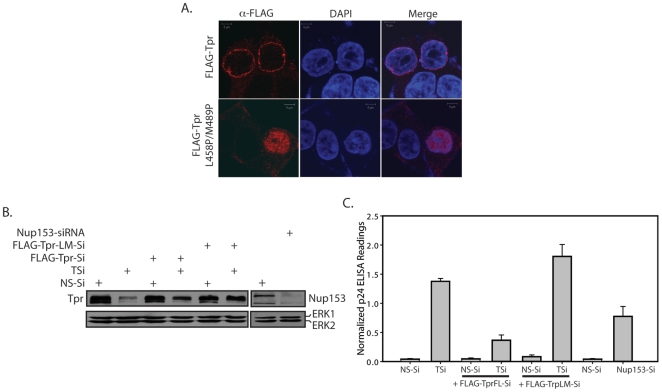
Localization of Tpr at the Nucleopore complex is important for the regulation of CTE mediated Export. (A) Immunofluorescence analysis of 293T cells transfected with Flag-Tpr or Flag-Tpr-L458P/M489P-Si constructs. (B) 293T cells were co-transfected with NS-Si or TSi or Nup153-siRNA, along with Gag/Pol-CTE and CMV- β-Gal reporter constructs, and 2 µg of Tpr-Si or Flag-Tpr-L458P/M489P-Si (Tpr-LM-Si) constructs. Western blot analysis indicating similar expression levels of Tpr-Si and Tpr-LM-Si constructs after depletion of endogenous Tpr. (C) p24 and β-Galactosidase levels in the whole cell extracts of each of the samples were analyzed. The p24 values were then adjusted for variations in β-Gal readings.

Nup153 has been demonstrated to interact with Tpr and the interaction is mediated through residues L458 and M489 in the N-terminal coiled-coil region of Tpr. The interaction has been shown to be required for the localization of Tpr [7]. The fact that we did not observe a cumulative effect upon Tpr and Nup153 double knockdown, and that Tpr-NM fragment rescued the phenotype, suggested that Nup153 mediated Tpr anchoring may be essential for modulating Gag/Pol-CTE RNA export. To test this, we generated siRNA resistant localization deficient mutant of Tpr (Tpr-L458P/M489P). As expected, FLAG-Tpr-Si was localized to the nuclear membrane (Fig. 10A). However, in agreement with the earlier reports [7], the localization of Tpr-L458P/M489P-Si mutant was observed to be intranuclear (Fig. 10A and S7A). As expected, when cells were transfected with siRNA resistant FLAG-Tpr-Si construct, we observed decreased p24 levels (Fig. 10B, Fig. S7B and S7C). Importantly, presence of siRNA resistant FLAG-Tpr-L458P/M489P-Si did not bring down the levels of normalized p24, though its expression was similar to FLAG-Tpr-Si (Fig. 10B and 10C). Taken together, these results provide compelling evidence that the localization of Tpr to the NPC is critical for regulating the export of intron containing RNA.

## Discussion

In the past few years, several groups have focused their research on the cellular functions of nucleoporins, including those that are localized to the nuclear basket region such as Tpr and Nup153. Tpr has been shown to activate mitotic spindle checkpoint [16] and its association with dynein complex was shown to be important for proper segregation of chromosomes during mitosis [17]. A recent study has shown that nucleoporin Tpr is important for establishing perinuclear heterochromatin exclusion zones [42]. These observations implicate a critical role for the human Tpr protein in modulating diverse functions. However, the role played by nucleoporin Tpr in the nucleocytoplasmic transport of macromolecules has not been comprehensively investigated. Studies performed with the yeast homolog of Tpr, Mlp1 reveals a role for the protein in the nuclear retention of unspliced RNA [30].

In mammalian cells, mRNA transcripts with unspliced introns are restricted to the nucleus. Retroviruses overcome this retention and export their full-length genomic RNA containing introns with the help of *cis*-acting elements like RRE (and transactivator protein Rev) or CTE [24], [43], [44]. We observed a significant enhancement of CTE function in Tpr depleted 293T cells, suggesting that Tpr plays a critical role in restricting the CTE-mediated export of intron-containing RNA. The fact that the protein transport, poly(A)^+^ mRNA distribution and the Rev-mediated unspliced RNA export remained unperturbed by Tpr knockdown, indicates that Tpr may specifically interact either directly or indirectly with the CTE-mRNP complexes in order to modulate their export through the NPC. Moreover, an increase in the nuclear CTE-RNA levels observed in the absence of Tpr also suggests that this nucleoporin may play an additional role in regulating the processing of these transcripts.

Sam68, Nxt1/p15 and Tap/Nxf1 proteins have been previously reported to play a role in enhancement of CTE function [26], [33], [34], [45]. The formation of Tap and Nxt heterodimers augments the binding of nucleoporins to Tap, and results in its increased nucleocytoplasmic shuttling [46]. Association of Nxt1 to Tap-CTE mRNA enhances the interaction of the mRNP complex to the NPC, resulting in its efficient export [47]. These proteins have also been shown to promote polyribosome association of intron-containing RNA, resulting in their efficient translation [34]. Since depletion of Tpr and expression of Sam68, Tap and Nxt1 regulate the export of unspliced RNA, we investigated the possibility of direct interactions between Tpr and Tap, Nxt1 or Sam68. While we observed an interaction between endogenous Tpr with immunoprecipitated ERK2 [22], we could not detect any interaction of Tpr with Sam68, Tap or Nxt1 proteins (Fig. S8). Tap/Nxf1 has been shown to have an effect on the export of mRNA from the nucleus in various model systems [37], [38], [39], [40]. In our study, upon partial depletion of Tap we observed a slight reduction in the p24 and β-GAL levels (Fig. 7A and 7B). However, when Tap was efficiently depleted (Fig. 7C–E), consistent with the previous findings, we observed a substantial reduction in β-GAL readings. On the contrary, depletion of Tap, or Tap and Tpr together, did not significantly alter the p24 levels over the levels observed upon Tpr depletion alone, suggesting that the change in Tpr levels is sufficient for the release of CTE-mRNP complexes into the cytosol. Further, we observed that the simultaneous depletion of Tpr and overexpression of Sam68 resulted in a synergistic increase in the export of unspliced RNA. These results indicate that the mode of regulating unspliced RNA export by Sam68 and Tpr are likely to be different. As has been shown earlier, Sam68 is most likely required for stabilizing the mRNA and for improved cytosolic utilization of unspliced RNA [26], while the function of Tpr is and may be mediated by either direct interaction, or through some unidentified RNA binding protein. In yeast, the RNA binding proteins Yra1p and Nab2p have been shown to be essential for the docking of mRNP complexes near the Mlp export gate located at the nuclear membrane [35]. A recent study demonstrated that depletion of a RNA endonuclease protein Swt1 resulted in nuclear poly (A)+ mRNA accumulation and enhanced the cytoplasmic leakage of unspliced pre-mRNAs in mlp1Δ and nup60Δ Mutants [48]. The homologs of Swt1 in humans, Smg5 and Smg6 proteins which were shown to play a role in nonsense- mediated mRNA decay [49], [50], [51]. Our preliminary data with the depletion of Smg5 and Smg6 proteins in Tpr knockdown cells to study the CTE dependent unspliced RNA export revealed no significant effect of these proteins (data not shown). Future studies would aimed at investigating the role of mammalian homologs of yeast proteins in regulating Tpr mediated unspliced RNA export.

FG repeat containing nucleoporins in the NPC are known to be required for transport of macromolecules across the nuclear pore [41], [52]. Members of the karyopherin/importin superfamily, the mRNA export receptor Mex67/Mtr2, and the Ran transporter Ntf2, have been shown to specifically interact with FG Nups [53], [54]. We did not detect increased p24 levels, when we depleted Nup98, Nup214, Nup358 and Nup50. Nup98 is a nucleoporin that has been shown to associate with the intranuclear Tpr filaments [11]. Powers, et al., have shown that the injection of monospecific polyclonal anit-Nup98 antibodies into the nucleus, inhibited export of multiple classes of RNA, including mRNA [55]. Nup98 has also been shown to interact with mRNA transport factors such as Rae1 and Tap/Mex67 [39], [56]. We observed that siRNA mediated depletion of Nup98 did not significantly affect either p24 or β-GAL readings (Fig. S5A and S5B), suggesting that Nup98 does not play a significant role in the export of unspliced RNA or mRNA. The differences detected in our observations could be due to the variance in the methods used for investigation. However, one needs to perform a systematic investigation using multiple methods to delineate the role played by Nup98 in mRNA export.

Interestingly, depletion of Nup153, an FG repeat containing nucleoporin that plays a critical role in various cellular processes including cell cycle progression [57], resulted in increased p24 levels. The overexpression of Nup153 has been shown to cause an accumulation of poly(A)^+^ mRNA in the nucleus [58], and the injection of antibodies against Nup153 were reported to block NES mediated export of proteins, U1 snRNA, 5sRNA and spliced mRNA [59]. The observed increase in p24 levels upon Nup153 depletion, though significant, was not as effective as that seen upon depletion of Tpr. A combined knockdown of Nup153 and Tpr did not show synergistic effect on the p24 levels, indicating that both of them most likely are functioning through the same pathway. In a previous study, Nup153 has been demonstrated to be required for anchoring Tpr to the NPC [7], and the L458 and M489 residues in the N-terminal region of Tpr have been shown to be necessary for their interaction. Taken together, the effect shown by Nup153 on CTE-mediated export may be due to the possibility that Nup153 depletion prevents or destabilizes NPC binding of Tpr, and demonstrates that the association of Tpr with the NPC is critical for its function. The results we obtained with localization-deficient Tpr mutant whereupon mislocalization of Tpr to the interior of the nucleus resulted in failure to regulate the RNA export, suggests that perinuclear localization of Tpr is an important step in regulating CTE mediated export.

While this manuscript was under preparation, Hammarskjold's group reported that Tpr regulates the export of mRNA with a *cis*- acting CTE element and that the transport of intron-containing mRNA's through the Crm1 pathway is unaffected by Tpr [60]. The authors also showed that rescue with siRNA resistant form of Tpr suppresses the effect on CTE mediated export and that the co-expression of Sam68 and Tpr-shRNA led to significant enhancement of p24 expression. These results corroborate our findings [60]. The authors suggest that the additive effect seen upon Sam68 over expression and Tpr knockdown, may be due to cross- talk between the two proteins, however, in our study we did not detect any direct interaction between Tpr and Sam68 (Fig. S8). Based on their experimental results Coyle et al also suggest that freshly formed Tpr molecules, which constitute minor but dynamic nuclear Tpr protein pools, which are involved in transcriptional regulation of CTE-mRNP complexes are sufficient for enhancement of p24 expression. The fact that we observe an increase in the nuclear CTE-mRNA levels upon Tpr depletion is in support of this hypothesis. The results of our experiments with Nup153 siRNA-mediated knockdown, as well as Tpr localization mutant studies, indicate that Tpr localized to the NPC plays a critical role in regulating unspliced RNA export. Apart from the regulatory role of Tpr in nuclear export, the authors report that depletion of Tpr leads to an increased association of CTE-mRNP complexes at the polyribosomal complexes, demonstrating a role for nucleoporin Tpr in the cytoplasm. However, Tpr depletion had no bearing on the stability of Gag/Pol protein in the cytosol. While our study does not address possible roles of Tpr in the cytoplasm, an increase in the nuclear CTE-mRNA pool upon Tpr depletion (Fig. 6) suggests a role for Tpr in the nucleoplasm. Taking together the findings of both studies, it appears the regulation of CTE-mediated unspliced RNA export is a complex and controlled phenomenon involving a plethora of proteins acting at various levels in the nucleus, of which nucleoporin Tpr is an important modulator. Future investigations would be directed towards identifying the key RNA binding proteins interacting with Tpr, and deciphering the precise mechanism by which Tpr modulates CTE-dependent unspliced RNA export.

## Materials and Methods

### Plasmid Constructs and siRNA Oligonucleotides

The siRNA oligonucleotides (Table S1) were purchased from either Dharmacon or Invitrogen. pCMV-Gag/Pol-CTE, pCMV-Gag/PR-RRE and pCMV-β-Gal constructs were kindly provided by Dr. Marie-Louise Hammarskjold Coyle [26] (University of Virginia), Dr. Hans-Georg Krausslich [33] (University of Hamburg) and Dr. Sagar Sengupta (National Institute of Immunology) respectively. HEK293T, COS-1 and HeLa cells were procured from ATCC, USA.

The construction of pcDNA3-FLAG-TprFL plasmid has been reported previously [22]. The Tpr-Si and Tpr-L458P/M489P clones were generated by overlapping PCR using specific primers. The Luc-CTE construct was generated by amplifying the 5′ Splice site region, and 3′ Splice site region, CTE region, and luciferase gene, and cloning them into pcDNA3.1 vector. FLAG-Rev and HA-Tap was generated by amplifying the coding regions and cloning them into pcDNA3-FLAG and pcDNA3-HA vectors, respectively. The HA-Sam68 clone was from Addgene (plasmid 17688; [61]).

### Cell Culture, Transfection and Generation of stable cell lines

HEK293T, COS-1 and HeLa cells were grown in DMEM supplemented with 10% fetal bovine serum (FBS). All transfections in HEK293T cells were performed in 6 well plates using calcium phosphate method. The transfections in HeLa, and COS-1 cells were performed by using Lipofectamine 2000 (Invitrogen) as per the manufacturer's recommendations. To analyze CTE- mediated export, cells were transfected with 250 ng pCMV-Gag/Pol-CTE, 100 ng pCMV- β-Gal and 1 µg of appropriate siRNA. In all the transfections, the final amount of plasmid DNA present in the transfection mix has been equalized by addition of appropriate amount of pCDNA3-FLAG vector. Cells were harvested 48 hours after transfection for further analysis unless mentioned otherwise. FLAG-Tpr and FLAG-Tpr-Si stable cell lines were generated by transfecting the constructs into HEK293T cells using Lipofectamine 2000 (Invitrogen). Twenty four hours after transfection, 10^4^ cells were plated in a 100 mm dish in medium containing 600 µg/ml Geneticin (Invitrogen), and incubated for 10 days. The colonies obtained were individually expanded and checked for expression of the introduced genes by Western Blot and indirect immunofluorescence analysis using anti-FLAG antibodies (Sigma).

### p24 ELISA and β-Galactosidase assay

Harvested cells were lysed with M2 Lysis buffer (50 mM Tris-HCl, 150 mM NaCl, 10% Glycerol, 1% Triton X-100, 0.5 mM EGTA, 0.5 mM EDTA) containing protease inhibitor cocktail (Roche). Lysates were clarified by high speed centrifugation. p24 expression levels were determined by sandwich ELISA using anti-mouse and anti-rabbit p24 monoclonal antibodies (obtained from AIDS Research and Reference Reagent program). β-Galactosidase (β-Gal) activity was assayed by incubating the lysate with 400 µg of *o*-nitrophenyl-β-D-galactopyranoside (ONPG) in Z buffer (40 mM Na_2_HPO_4_.7H_2_O, 60 mM NaH_2_PO_4_.H_2_O, 10 mM KCl, 1 mM MgSO_4_.7H_2_O) for 15 to 30 minutes and measuring absorbance at 420 nm. The p24 readings obtained from ELISA were then normalized with respect to β-Galactosidase readings. The data presented in the figures are average values obtained from three independent transfections; the error bars represent the standard deviation.

### Immunoblotting and Immunofluorescence

The mouse monoclonal antibody against Tpr (ab58344), anti-Nup153, anti-Nup98, anti-Nup214, anti-Sam68 and anti-Tap antibodies were from Abcam, anti-Nxt-1 and anti-cyclinA antibodies were from Santa Cruz Biotechnology. The anti-53BP1 antibody was kindly provided by Dr. Sagar Sengupta, anti-Erk2, anti-LaminA/C, and anti-Tubulin antibodies were purchased from Cell Signalling Technology. Anti-Nup358 antibody was kindly provided by Dr. Jomon Joseph. All secondary antibodies were obtained from Jackson Immuno Research Laboratories. Typically, 50 µg of the lysates were analyzed in Western Blot analysis after resolution on 10% SDS-PAGE. Immunofluorescence analysis was carried out with cells fixed with 4% paraformaldehyde, followed by permeabilization with 0.1% Triton X-100 for 10 minutes. Blocking was carried out with 10% normal chicken serum (NCS) at 4°C (12 hours), followed by incubation with appropriate antibodies. Coverslips were mounted using mounting medium containing DAPI (Vectashield) and visualized with the help of Carl Zeiss Axiovision LSM 510 Meta confocal microscope.

### Protein Transport and mRNA export Assays

The HeLa cell line stably expressing a chimeric Rev-GFP-Glucocorticoid Receptor protein (RGG) [29] was kindly provided by Dr. Jomon Joseph. Two 60 mm dishes of the above cells (5×10^5^ cells/dish) were transfected with 4 µg of NS-Si or TSi. 24 hours post-transfection, cells were trypsinized and plated on to coverslips (10^5^ cells/coverslip) and allowed to recover for 24 hours. We monitored the import of chimeric GFP by fixing the cells with 4% paraformaldehyde at different times post dexamethasone treatment. To study nuclear export, the cells treated with 1 µM dexamethasone for 60 minutes were washed to remove the hormone, and were replenished with fresh medium for different time intervals, as indicated.

An FITC-conjugated oligod(T) 23mer probe was procured from *Integrated DNA Technologies (USA)*. Fluorescence in situ hybridization was performed with this probe to analyze poly (A)^+^ mRNA export in HEK293T cells. 48 hours after siRNA transfection to induce Tpr knockdown, the cells (grown on coverslips) were fixed with 4% paraformaldehyde and permeabilized with 0.2% Triton X-100. The cells were incubated in a moist chamber for 3 hours at 37°C with the hybridization mix containing 20× SSC, 50% dextran sulphate, 1 µg/µl yeast tRNA and 1 pmol/µl of the labelled probe. Stringent post hybridization washes were done with 4× SSC and 0.1% Tween 20. Blocking was carried out with 10% normal chicken serum (NCS), followed by incubation with appropriate antibodies.

### RNA Isolation and qRT-PCR

HEK293T cells were harvested 48 hours after transfection with either NS-Si or TSi. Cells were lysed in Hypotonic Lysis Buffer (20 mM HEPES-NaOH, 2 mM EGTA and 2 mM MgCl_2_) for 10 min and subsequently centrifuged at 1200 r.p.m to sediment the nuclei and to obtain the cytoplasmic fraction as the supernatant. The nuclear pellet was washed once in the Cell fractionation buffer (PARIS Kit, Ambion). The Nuclear lysis and preparation of RNA from both cytosolic and nuclear extracts was performed using the PARIS kit according to manufacturer's recommendations. Synthesis of cDNA using an oligo (dT) primer was carried out by RETROscript Kit (Ambion). This cDNA was used for real time quantitative PCR analysis of Gag-Pol-CTE and *ERK2* (reference) genes using gene specific primers. The results obtained from real-time PCR data are represented as CT values, where CT is defined as the threshold cycle number at which there is a detection of amplified product. The average CT was calculated for both the target gene (Gag-Pol-CTE or Luc-CTE) and *ERK2* (Control) and the ΔCT was determined as (the mean of the triplicate CT values for the CTE gene) minus (the mean of the triplicate CT values for *ERK2*). The ΔΔCT represents the difference between the Tpr knockdown cells and Control cells, as calculated by the formula ΔΔCT = (ΔCT of Tpr Knockdown cells - ΔCT of control cells). The N-fold differential expression of the target gene (Gag-Pol-CTE or Luc-CTE) in Tpr depleted cells versus the control cells transfected with NS-Si, was expressed as 2^−ΔΔCT^ [62].

## Supporting Information

Figure S1 **Role of Tpr in Rev dependent and CTE mediated unspliced RNA export.** (A) Gag/PR-RRE reporter construct along with Flag-Rev and CMV- β-Gal plasmids were co-transfected with various Tpr siRNA oligos, and the lysates were assayed for p24 expression 48 hours later. (B) β-Galactosidase levels in each of the samples were assayed. Bars represent the mean of values obtained and the error bars represent the standard deviation (s.d) of values obtained from three independent transfections. The corresponding normalized p24 values (with respect to β-Gal) are represented in Figure 3B. (C) p24 ELISA readings of HEK293T cell extracts depicting elevated levels of Gag/Pol protein cleavage products in cells harvested 48 hours after transfection with Gag/Pol-CTE reporter construct, CMV- β-Gal and different Tpr siRNA's. (D) The β-Galactosidase activity is estimated in each of the samples. The corresponding normalized p24 values are represented in Figure 3D.
(TIF)

Figure S2 **Tpr depletion causes enhancement of CTE function in mammalian cells.** (A and B) HEK293T, COS-1 and HeLa cells were transfected with either NS-Si or TSi and Gag/Pol-CTE and CMV- β-Gal reporter construct and the lysates were assayed for p24 (Panel A) and β-Gal expression (Panel B) 48 hours after the transfection. Bars represent the mean of values and the error bars represent the s.d. of values obtained from three independent transfections. The corresponding normalized p24 values are represented in Figure 4B. (C) HEK293T cells were co-transfected with NS-Si or Tsi and CTE-Luc and CMV- β-Gal constructs, and the luciferase activity in the lysates was determined 48 hours post transfection. (D) β-Galactosidase levels in the samples was assayed. The corresponding normalized luciferase readings are represented in Figure 4E.
(TIF)

Figure S3 **Reduction in p24 levels is observed upon rescue with siRNA resistant clone of Tpr.** (A) HEK293T cells transiently transfected with Flag-Tpr and Flag-Tpr-Si constructs. (B and C) Flag-Tpr or FLAG-Tpr-Si constructs were tansfected into HEK293T cells along with NS-Si or TSi, and Gag/Pol-CTE and CMV-β-Gal reporter constructs. The p24 expression (Panel B) and β-Galactosidase levels (Panel C) in each of the samples were analyzed. The corresponding normalized p24 values are represented in Figure 5D. (D and E) HEK293T cells stably expressing Flag-Tpr or Flag-Tpr-Si were transfected with NS-Si or TSi together with Gag/Pol-CTE and CMV- β-Gal plasmids. The cell lysates were assayed for p24 (Panel D) and β-Gal expression (Panel E). The corresponding normalized p24 values are represented in Figure 5G.
(TIF)

Figure S4 **Regulation of unspliced RNA export in Tpr knockdown cells upon the combined depletion or overexpression of Tap/Nxf1 and Sam68 proteins.** (A and B) Cells were transfected with 1 µg of siRNA against either Sam68 or Tap/Nxf1 along with NS-Si or TSi. The cells were replated the next day and 24 hours after replating, the cells were transfected once again with siRNA oligos and the reporter constructs. 48 hours post-transfection, reporter gene expression from transfected Gag/Pol-CTE (Panel A) and CMV- β-Gal (Panel B) constructs were assayed. The corresponding normalized values are represented in Figure 7B. (C and D) After 48 hours of treatment with NS-Si and TSi, HEK293T cells were re-transfected with the same siRNA oligos along with Gag/Pol-CTE and CMV- β-Gal plasmids and HA-Sam68 or HA-Tap/Nxf1constructs. The lysates thus obtained were analyzed for the amounts of p24 (Panel C) and β-Gal expression (Panel D). Data represents the average of values and the error bars correspond to the s.d. obtained from three independent transfections. The corresponding normalized values are represented in Figure 8B.
(TIF)

Figure S5 **Depletion of Nup153 also has an effect on CTE dependent export.** (A and B) HEK293T cells were transfected with siRNA's against different nucleoporins together with Gag/Pol-CTE and CMV- β-Gal constructs. 48 hours post transfection, the expression of p24 (Panel A) and β-Gal (Panel B) were estimated in the lysates. The corresponding normalized values are represented in Figure 9B. (C and D) Cells were transfected with siRNA's against Nup153 or Tpr or Nup153+Tpr together with Gag/Pol-CTE and CMV- β-Gal constructs. p24 levels (Panel C) and β-Galactosidase readings (Panel D) observed in cells transfected with Gag/Pol-CTE and CMV- β-Gal reporter constructs along with TSi and/or Nup153-siRNA. The corresponding normalized values are represented in Figure 9D.
(TIF)

Figure S6 **The N-terminal region of Tpr is necessary for the regulation of CTE mediated unspliced RNA export.** (A) Schematic representation of various Tpr deletion fragments. (B) Immunoblot depicting the expression of various Flag-tagged Tpr deletion fragments in cells devoid of endogenous Tpr. (C and D) p24 levels (Panel C) and β-Galactosidase readings (Panel D) observed in cells transfected with Gag/Pol-CTE and CMV- β-Gal reporter constructs along with TSi and rescued with different deletion constructs of the protein. (E) Normalized p24 ELISA readings in HEK293T cells treated with TSi for 48 hours and rescued with different deletion constructs of the protein. (F) Immunofluorescence microscopy of the cells transfected with different deletion constructs of Tpr.
(TIF)

Figure S7 **Rescue with localization deficient mutant of Tpr does not result in the reduction in p24 levels.** (A) HEK293T cells transiently transfected with Flag-Tpr-L458P/M489P-Si construct. (B and C) Cells were co-transfected with NS-Si or TSi or Nup153-siRNA along with Gag/Pol-CTE and CMV- β-Gal reporter constructs and Tpr-Si or siRNA resistant localization mutant of Tpr (Tpr- L458P/M489P-Si). 48 hours post transfection, the amount of p24 and β-Gal expression was estimated in the lysates. The corresponding normalized values are represented in Figure 10C.
(TIF)

Figure S8 **Nucleoporin Tpr does not interact with Tap/Nxf1 and Sam68 proteins.** (A) Cells were transfected with HA-ERK2, HA-Sam68, HA-Nxt/p15 or HA-Tap/Nxf1 constructs. 24 hours post transfection, cells were lysed, and the lysates were immunoprecipitated with HA-antibodies. The immunoblots were probed with anti-HA and anti-Tpr antibodies to determine the interactions between Tpr and HA-tagged proteins. Co-immunoprecipitation of endogenous Tpr along with HA-ERK2 validates the approach.
(TIF)

Table S1 **Sequence of siRNA oligonucleotides used in the study.**
(DOC)
