# Supplementary figures and images for: Localization of Nucleoporin Tpr to the Nuclear Pore Complex Is Essential for Tpr Mediated Regulation of the Export of Unspliced RNA
Source: PLoS One. 2012 Jan 13;7(1):e29921. doi: 10.1371/journal.pone.0029921 (PMC3258255; doi:10.1371/journal.pone.0029921)

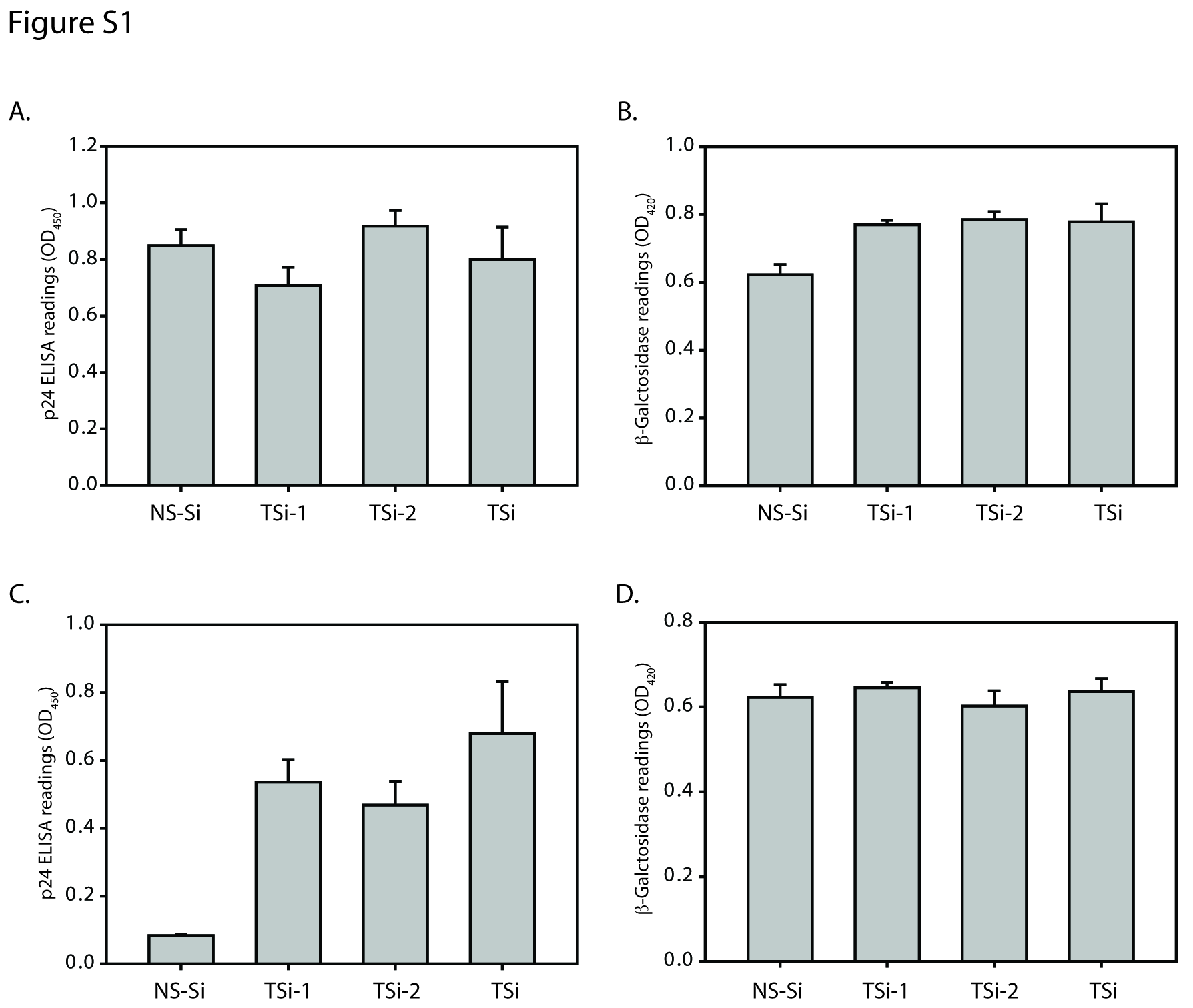

Supplement: Figure S1 — Role of Tpr in Rev dependent and CTE mediated unspliced RNA export. (A) Gag/PR-RRE reporter construct along with Flag-Rev and CMV- β-Gal plasmids were co-transfected with various Tpr siRNA oligos, and the lysates were assayed for p24 expression 48 hours later. (B) β-Galactosidase levels in each of the samples were assayed. Bars represent the mean of values obtained and the error bars represent the standard deviation (s.d) of values obtained from three independent transfections. The corresponding normalized p24 values (with respect to β-Gal) are represented in Figure 3B. (C) p24 ELISA readings of HEK293T cell extracts depicting elevated levels of Gag/Pol protein cleavage products in cells harvested 48 hours after transfection with Gag/Pol-CTE reporter construct, CMV- β-Gal and different Tpr siRNA's. (D) The β-Galactosidase activity is estimated in each of the samples. The corresponding normalized p24 values are represented in Figure 3D. (TIF) [file pone.0029921.s001.tif]

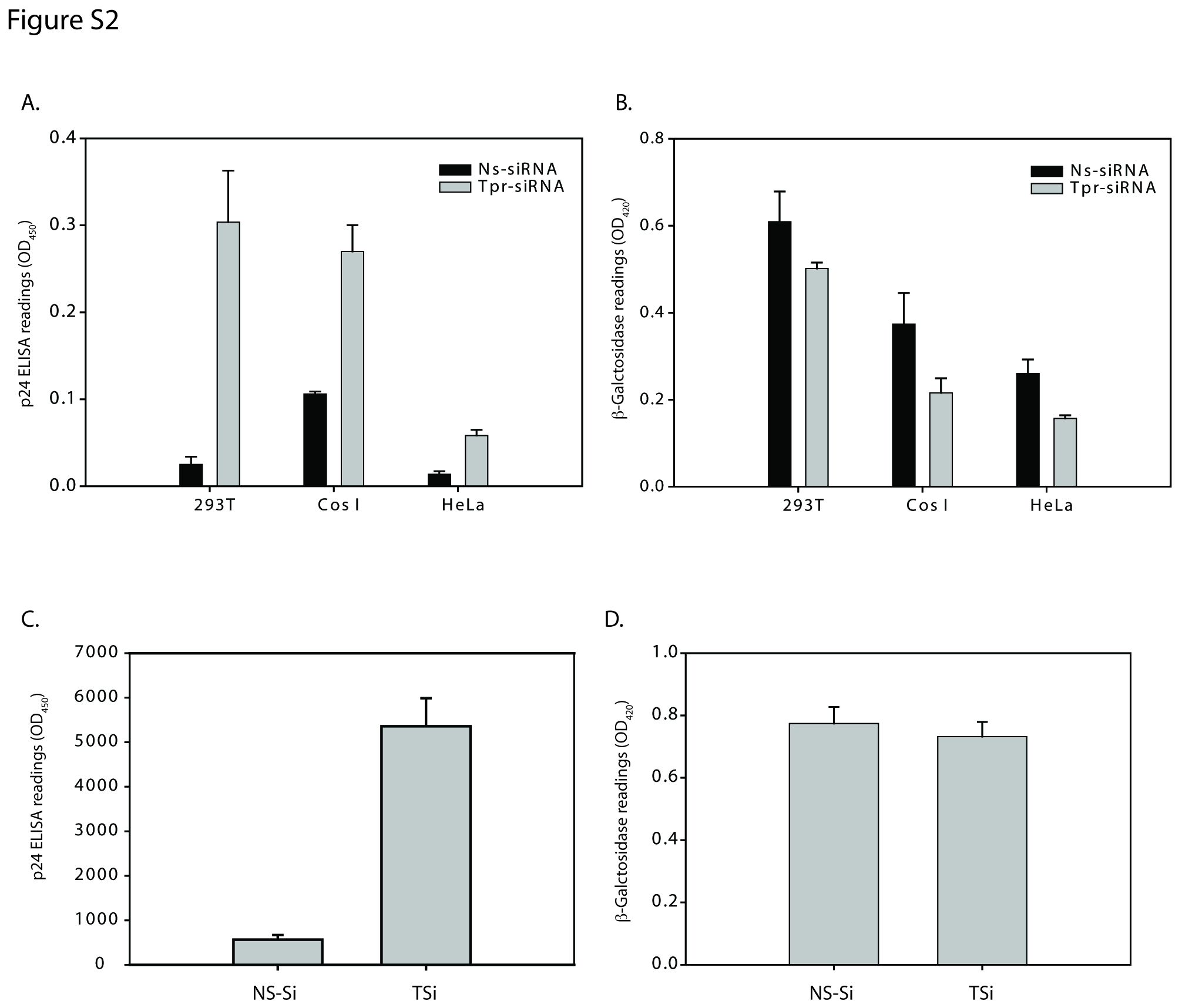

Supplement: Figure S2 — Tpr depletion causes enhancement of CTE function in mammalian cells. (A and B) HEK293T, COS-1 and HeLa cells were transfected with either NS-Si or TSi and Gag/Pol-CTE and CMV- β-Gal reporter construct and the lysates were assayed for p24 (Panel A) and β-Gal expression (Panel B) 48 hours after the transfection. Bars represent the mean of values and the error bars represent the s.d. of values obtained from three independent transfections. The corresponding normalized p24 values are represented in Figure 4B. (C) HEK293T cells were co-transfected with NS-Si or Tsi and CTE-Luc and CMV- β-Gal constructs, and the luciferase activity in the lysates was determined 48 hours post transfection. (D) β-Galactosidase levels in the samples was assayed. The corresponding normalized luciferase readings are represented in Figure 4E. (TIF) [file pone.0029921.s002.tif]

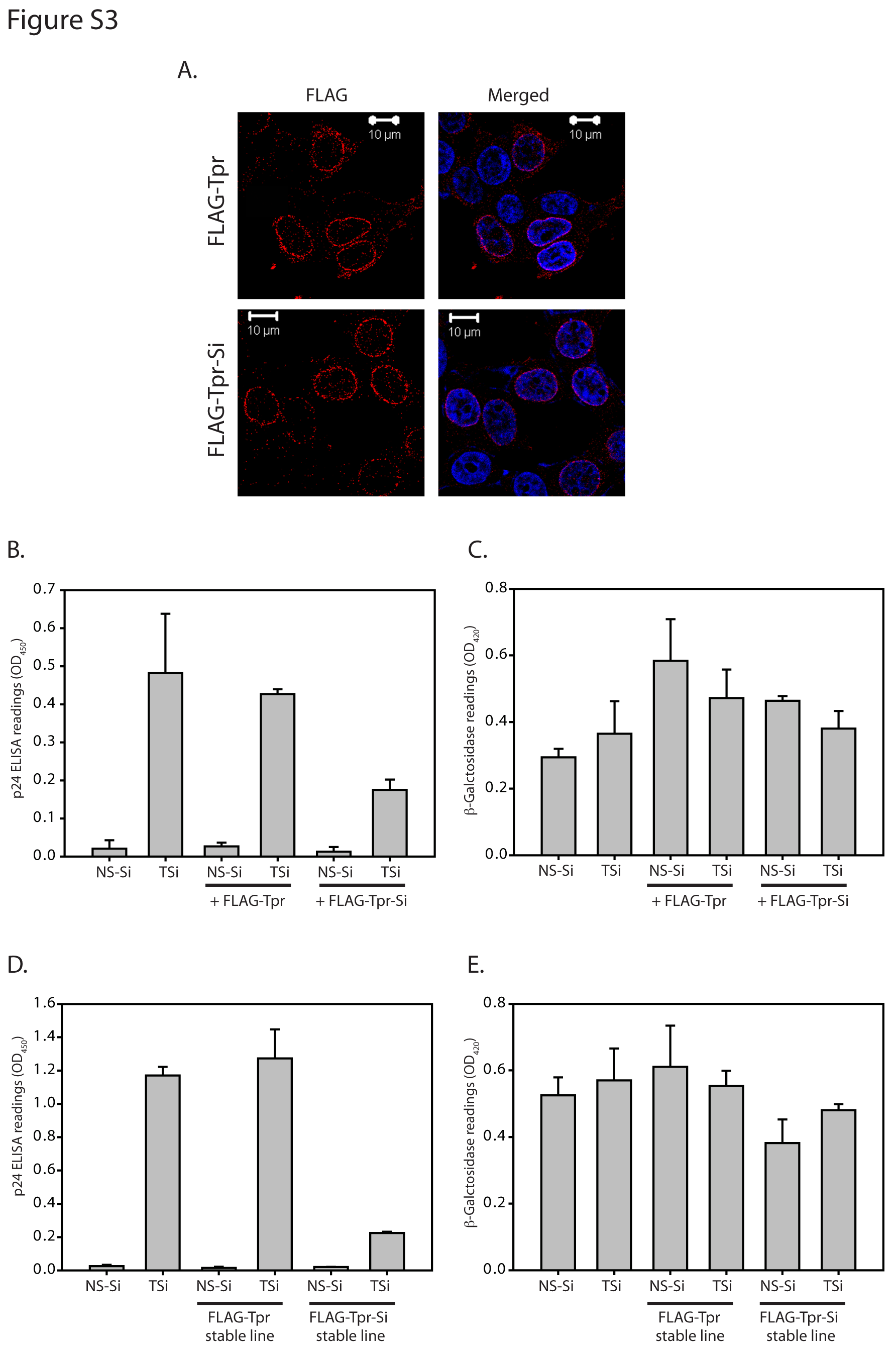

Supplement: Figure S3 — Reduction in p24 levels is observed upon rescue with siRNA resistant clone of Tpr. (A) HEK293T cells transiently transfected with Flag-Tpr and Flag-Tpr-Si constructs. (B and C) Flag-Tpr or FLAG-Tpr-Si constructs were tansfected into HEK293T cells along with NS-Si or TSi, and Gag/Pol-CTE and CMV-β-Gal reporter constructs. The p24 expression (Panel B) and β-Galactosidase levels (Panel C) in each of the samples were analyzed. The corresponding normalized p24 values are represented in Figure 5D. (D and E) HEK293T cells stably expressing Flag-Tpr or Flag-Tpr-Si were transfected with NS-Si or TSi together with Gag/Pol-CTE and CMV- β-Gal plasmids. The cell lysates were assayed for p24 (Panel D) and β-Gal expression (Panel E). The corresponding normalized p24 values are represented in Figure 5G. (TIF) [file pone.0029921.s003.tif]

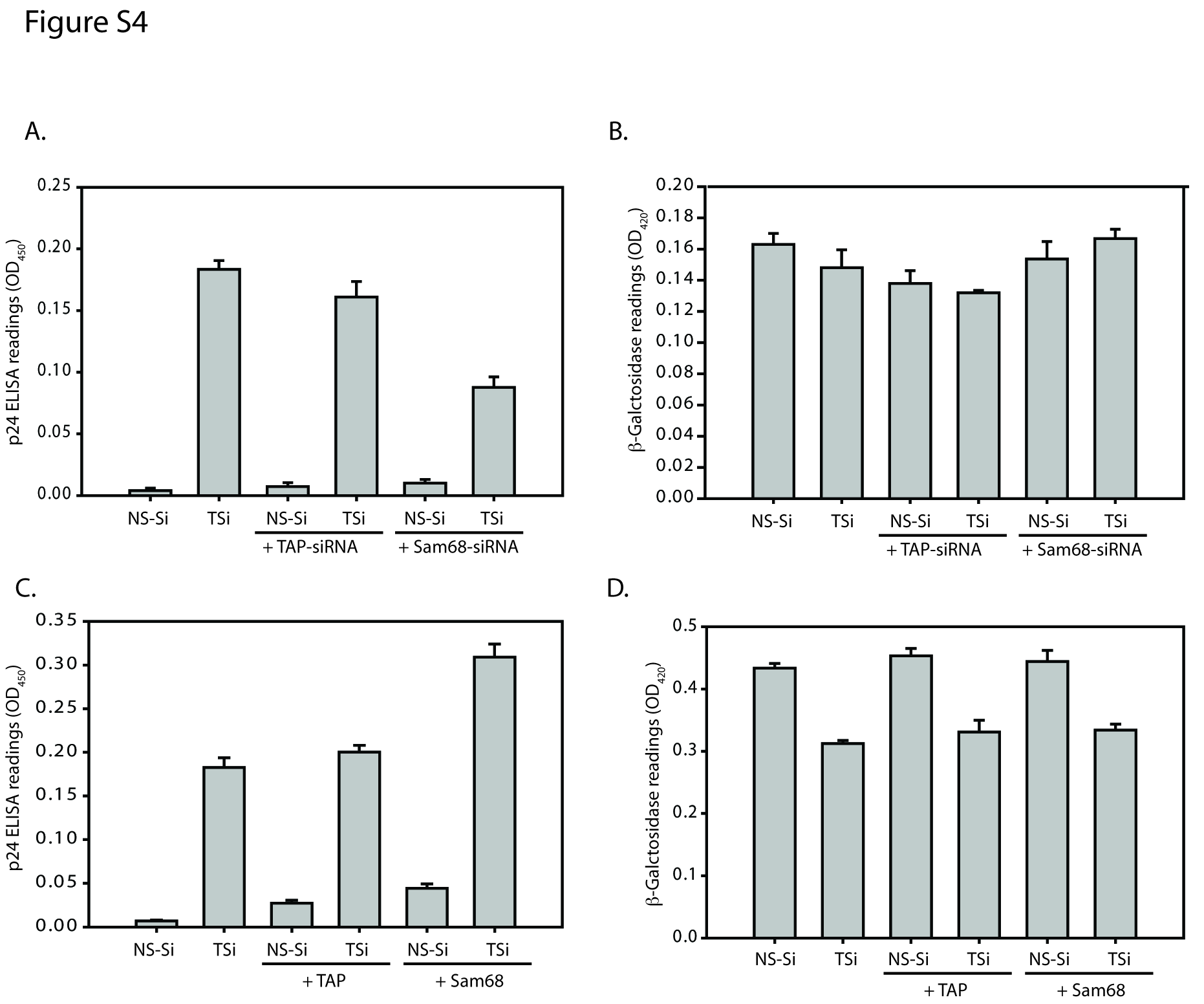

Supplement: Figure S4 — Regulation of unspliced RNA export in Tpr knockdown cells upon the combined depletion or overexpression of Tap/Nxf1 and Sam68 proteins. (A and B) Cells were transfected with 1 µg of siRNA against either Sam68 or Tap/Nxf1 along with NS-Si or TSi. The cells were replated the next day and 24 hours after replating, the cells were transfected once again with siRNA oligos and the reporter constructs. 48 hours post-transfection, reporter gene expression from transfected Gag/Pol-CTE (Panel A) and CMV- β-Gal (Panel B) constructs were assayed. The corresponding normalized values are represented in Figure 7B. (C and D) After 48 hours of treatment with NS-Si and TSi, HEK293T cells were re-transfected with the same siRNA oligos along with Gag/Pol-CTE and CMV- β-Gal plasmids and HA-Sam68 or HA-Tap/Nxf1constructs. The lysates thus obtained were analyzed for the amounts of p24 (Panel C) and β-Gal expression (Panel D). Data represents the average of values and the error bars correspond to the s.d. obtained from three independent transfections. The corresponding normalized values are represented in Figure 8B. (TIF) [file pone.0029921.s004.tif]

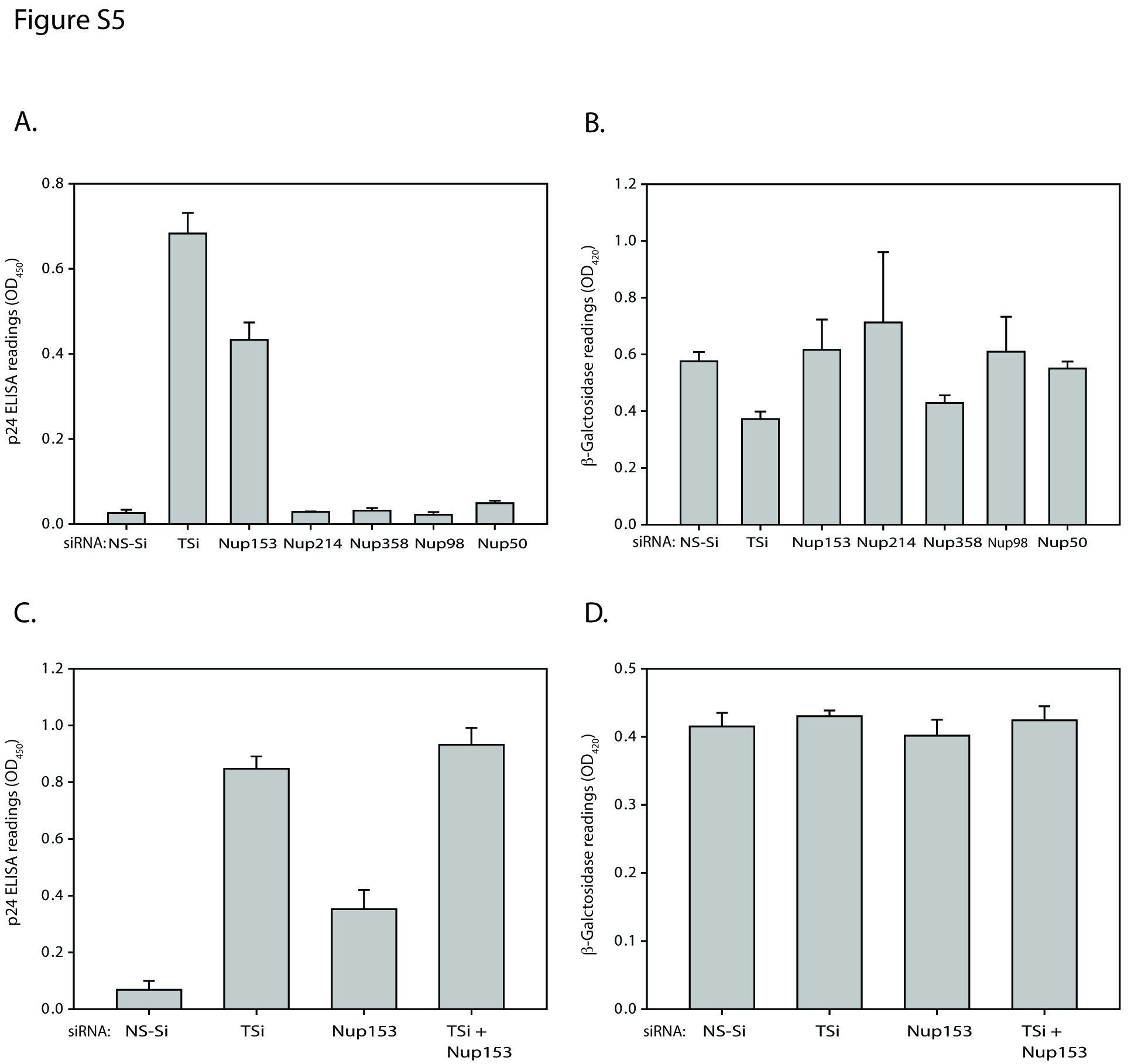

Supplement: Figure S5 — Depletion of Nup153 also has an effect on CTE dependent export. (A and B) HEK293T cells were transfected with siRNA's against different nucleoporins together with Gag/Pol-CTE and CMV- β-Gal constructs. 48 hours post transfection, the expression of p24 (Panel A) and β-Gal (Panel B) were estimated in the lysates. The corresponding normalized values are represented in Figure 9B. (C and D) Cells were transfected with siRNA's against Nup153 or Tpr or Nup153+Tpr together with Gag/Pol-CTE and CMV- β-Gal constructs. p24 levels (Panel C) and β-Galactosidase readings (Panel D) observed in cells transfected with Gag/Pol-CTE and CMV- β-Gal reporter constructs along with TSi and/or Nup153-siRNA. The corresponding normalized values are represented in Figure 9D. (TIF) [file pone.0029921.s005.tif]

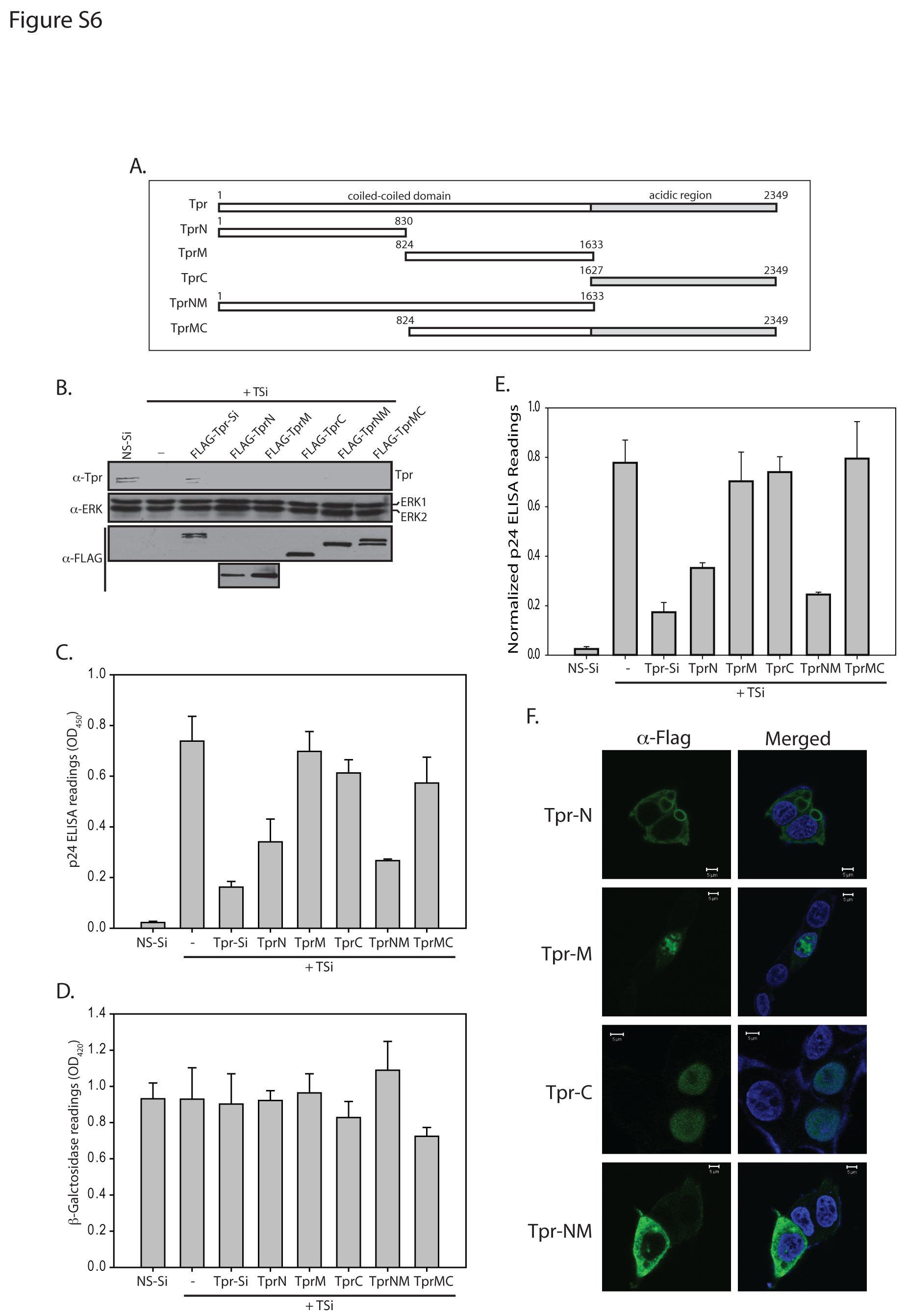

Supplement: Figure S6 — The N-terminal region of Tpr is necessary for the regulation of CTE mediated unspliced RNA export. (A) Schematic representation of various Tpr deletion fragments. (B) Immunoblot depicting the expression of various Flag-tagged Tpr deletion fragments in cells devoid of endogenous Tpr. (C and D) p24 levels (Panel C) and β-Galactosidase readings (Panel D) observed in cells transfected with Gag/Pol-CTE and CMV- β-Gal reporter constructs along with TSi and rescued with different deletion constructs of the protein. (E) Normalized p24 ELISA readings in HEK293T cells treated with TSi for 48 hours and rescued with different deletion constructs of the protein. (F) Immunofluorescence microscopy of the cells transfected with different deletion constructs of Tpr. (TIF) [file pone.0029921.s006.tif]

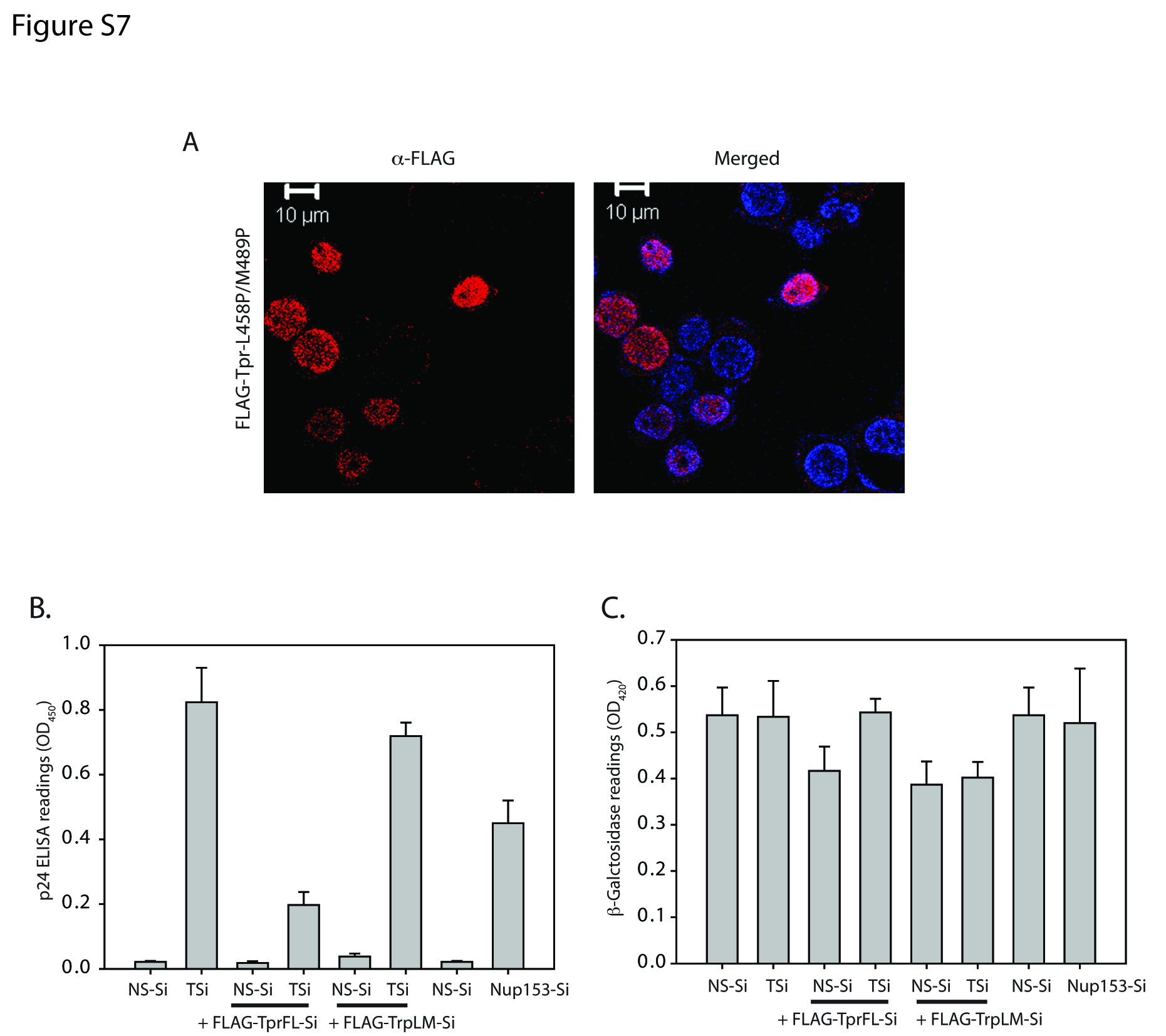

Supplement: Figure S7 — Rescue with localization deficient mutant of Tpr does not result in the reduction in p24 levels. (A) HEK293T cells transiently transfected with Flag-Tpr-L458P/M489P-Si construct. (B and C) Cells were co-transfected with NS-Si or TSi or Nup153-siRNA along with Gag/Pol-CTE and CMV- β-Gal reporter constructs and Tpr-Si or siRNA resistant localization mutant of Tpr (Tpr- L458P/M489P-Si). 48 hours post transfection, the amount of p24 and β-Gal expression was estimated in the lysates. The corresponding normalized values are represented in Figure 10C. (TIF) [file pone.0029921.s007.tif]

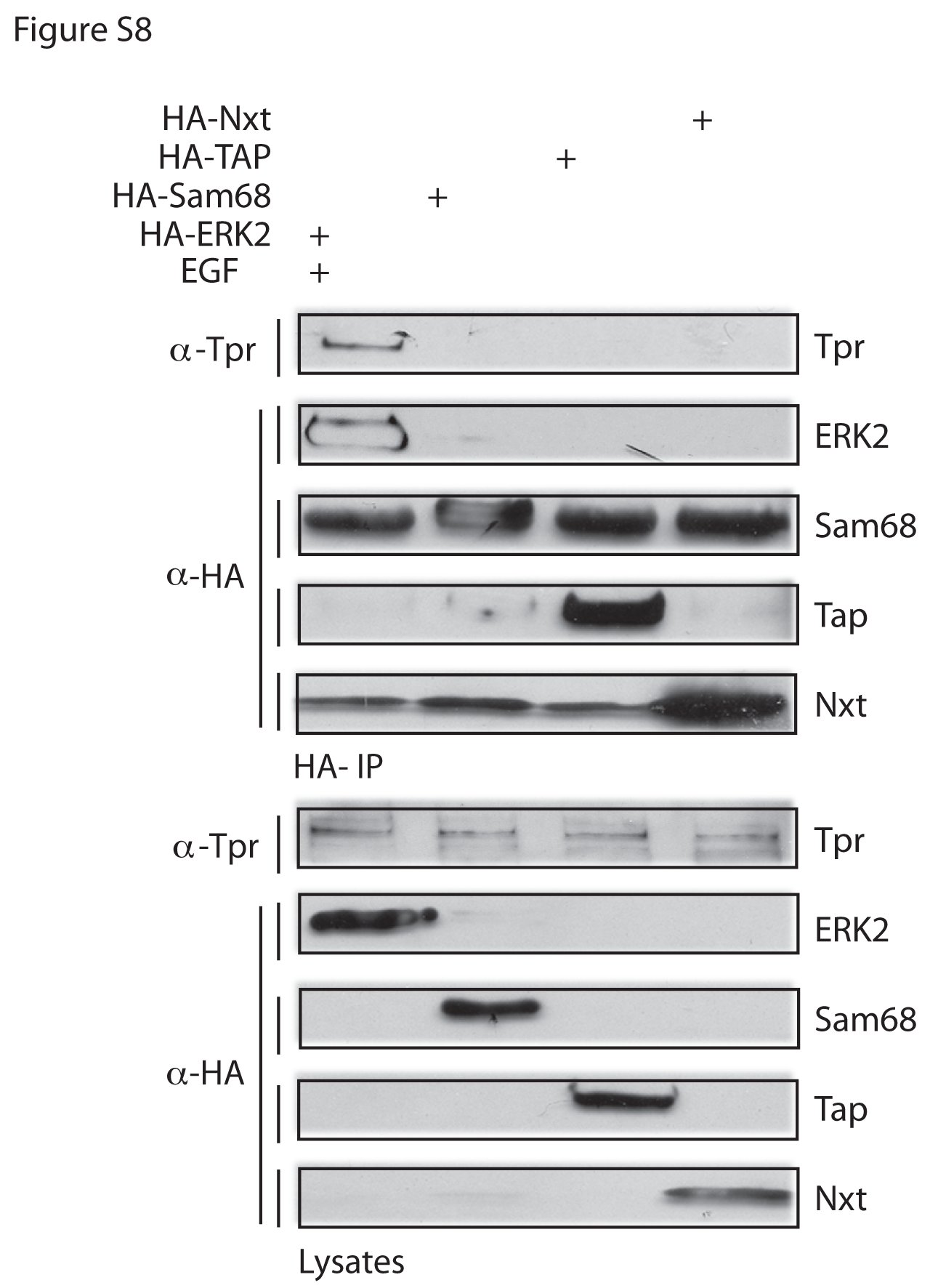

Supplement: Figure S8 — Nucleoporin Tpr does not interact with Tap/Nxf1 and Sam68 proteins. (A) Cells were transfected with HA-ERK2, HA-Sam68, HA-Nxt/p15 or HA-Tap/Nxf1 constructs. 24 hours post transfection, cells were lysed, and the lysates were immunoprecipitated with HA-antibodies. The immunoblots were probed with anti-HA and anti-Tpr antibodies to determine the interactions between Tpr and HA-tagged proteins. Co-immunoprecipitation of endogenous Tpr along with HA-ERK2 validates the approach. (TIF) [file pone.0029921.s008.tif]
