# Supplementary material for: Localization of Nucleoporin Tpr to the Nuclear Pore Complex Is Essential for Tpr Mediated Regulation of the Export of Unspliced RNA
Source: PLoS One. 2012 Jan 13;7(1):e29921. doi: 10.1371/journal.pone.0029921 (PMC3258255; doi:10.1371/journal.pone.0029921)
Supplement: Table S1 — Sequence of siRNA oligonucleotides used in the study. (DOC) [file pone.0029921.s009.doc]

Table S1

| **Name of the siRNA** | **Sequence** |
| --- | --- |
| Tpr-siRNA (TSi) | GCACAACCAGGATAAGGTTA |
| Tpr-siRNA1 (TSi-1) | GAAGAAGUGCGUAAGAAUA |
| Tpr-siRNA2 (TSi-2) | GGCAUACACUUACUAGAAA |
| Nup153-siRNA | GAUAGGAGUGGGAUAGAUA |
| Nup214-siRNA | UCAAAUACCUCUAACCUAU |
| Nup358-siRNA | GCGAAGUGAUGAUAUGUUU |
| Nup98-siRNA | GAACAACCAACCUAAGAUU |
| Nup50-siRNA | CCAAAGUAGUAGUUACCGA |
| Sam68-siRNA | GCACCCAUAUGGACGUUAU |
| Tap-siRNA | GGGAAGUCGUACAGCGAAC |
| Tap-siRNA-2 | GCGCCAUUCGCGAACGAUU |
| Tap-siRNA-SP | CGAUGAUGAACGCGUUAAU  AAUUGAAGUCUGAGCGGGA  GGGAAGUCGUACAGCGAAC  GCGCCAUUCGCGAACGAUU |
